# A mutated glycosaminoglycan-binding domain functions as a novel probe to selectively target heparin-like epitopes on tumor cells

**DOI:** 10.1016/j.jbc.2022.102609

**Published:** 2022-10-17

**Authors:** Yingying Xu, Liran Shi, Yong Qin, Xunyi Yuan, Xu Wang, Qingdong Zhang, Lin Wei, Min Du, Yi Liu, Min Yuan, Xiangyu Xu, Ruiqing Cheng, Ruyi Zou, Wenshuang Wang, Fuchuan Li

**Affiliations:** 1 National Glycoengineering Research Center and Shandong Provincial Key Laboratory of Carbohydrate Chemistry and Glycobiology, Shandong University, Qingdao, Shandong province, People’s Republic of China; 2 CSPC Megalith Biopharmaceutical Co, Ltd, Antibody Discovery Laboratory of New Drug Development, shijiazhuang, Hebei province, People’s Republic of China; 3 Department of General Surgery, Qilu Hospital, Shandong University, Qingdao, Shandong province, People’s Republic of China; 4 School of Life Science and Technology, Weifang Medical University, Weifang, Shandong province, People’s Republic of China; 5 Department of Gastroenterology, The Affiliated Hospital of Qingdao University, Qingdao, Shandong province, People’s Republic of China

**Keywords:** glycosaminoglycan, heparin, heparin-like epitope, probe, tumor targeting, CS, chondroitin sulfate, DS, dermatan sulfate, FBS, fetal bovine serum, FITC, fluorescein isothiocyanate, GAG, glycosaminoglycan, HCC, hepatocellular carcinoma, Hep, heparin, HRP, horseradish peroxidase, HS, heparan sulfate, PI, isoelectric point, scFv, single-chain variable fragment

## Abstract

The high heterogeneity and mutation rate of cancer cells often lead to the failure of targeted therapy, and therefore, new targets for multitarget therapy of tumors are urgently needed. Aberrantly expressed glycosaminoglycans (GAGs) have been shown to be involved in tumorigenesis and are promising new targets. Recently, the GAG-binding domain rVAR2 of the *Plasmodium falciparum* VAR2CSA protein was identified as a probe targeting cancer-associated chondroitin sulfate A-like epitopes. In this study, we found that rVAR2 could also bind to heparin (Hep) and chondroitin sulfate E. Therefore, we used rVAR2 as a model to establish a method based on random mutagenesis of the GAG-binding protein and phage display to identify and optimize probes targeting tumor GAGs. We identified a new probe, VAR2HP, which selectively recognized Hep by interacting with unique epitopes consisting of a decasaccharide structure that contains at least three HexA2S(1–4)GlcNS6S disaccharides. Moreover, we found that these Hep-like epitopes were overexpressed in various cancer cells. Most importantly, our *in vivo* experiments showed that VAR2HP had good biocompatibility and preferentially localizes to tumors, which indicates that VAR2HP has great application potential in tumor diagnosis and targeted therapy. In conclusion, this study provides a strategy for the discovery of novel tumor-associated GAG epitopes and their specific probes.

Targeted therapies have been recognized as the most promising therapeutic strategy for cancers because they can directly alter cell signaling events through the use of inhibitors and indirectly deliver antineoplastic drugs to cancer cells through tumor-targeting molecules (1, 2, 3). Currently, the majority of molecules targeted by therapeutic ligands are cellular proteins, such as human epidermal growth factor receptor 2, which is used for the targeted therapy of HER2-positive breast cancer (4, 5), and folate receptor, which is used for the treatment of folate receptor–overexpressing cancers (6, 7, 8). However, the high heterogeneity and mutation of cancer cells are often the main reasons for the failure of targeted therapy (9). Thus, the identification of tumor-associated neoantigens/neoepitopes is urgently needed for the diagnosis and multitargeted therapy of cancers (10).

Glycosaminoglycans (GAGs), linear negatively charged heteropolysaccharides composed of repeating hexosamine-containing disaccharide units, are ubiquitously expressed in all mammalian tissues. GAGs are classified into four categories according to the type of disaccharide composition and linkage between disaccharide units: chondroitin sulfate (CS)/dermatan sulfate (DS), heparin (Hep)/heparan sulfate (HS), hyaluronan, and keratan sulfate. GAGs participate in various physiological and pathological processes including tumorigenesis (11, 12, 13, 14). Aberrantly expressed GAGs in structure and/or quantity are involved in the proliferation, invasion, adhesion, and migration of tumor cells and therefore have the potential to be used as targets for the diagnosis and therapy (15, 16).

Some GAG-based neoantigens/neoepitopes have been identified as promising therapeutic targets in cancers. For example, a HS motif (GlcNS6S-IdoA2S)_3_ recognized by the single-chain variable fragment (scFv) antibody NS4F5 is upregulated in ovarian cancer (17). Another scFv antibody, HS20, inhibits the proliferation of hepatocellular carcinoma (HCC) cells *in vitro* and *in vivo* by binding to the HS chains of glypican-3 to block Wnt3a/β-catenin signaling, which recognizes HS structures containing IdoA2S and GlcNS6S (18, 19, 20). In addition, a CS-E-like epitope rich in GlcAβ1-3GalNAc(4,6-*O*-disulfate) has been detected in the mouse Lewis lung carcinoma cell line LM66-H11 with high metastatic potential (21), murine LM8G7 osteosarcoma cells (22) and ovarian adenocarcinomas by the scFv antibody GD3G7 (23), which has been shown to be a potential biomarker for the diagnosis and therapy of malignant ovarian tumors (24, 25). Notably, all these studies were based on the development and application of anti-GAG antibodies selected from established semisynthetic scFv libraries (26). Considering the high complexity of GAG chains, however, it is necessary to develop new approaches to specifically screen novel probes and identify their GAG-based epitopes in cancer cells.

Recently, a CS-A-like epitope rich in GlcAβ1-3GalNAc(4-*O*-sulfate), which is exclusively expressed in the placenta, has been found in a various of malignant cells and can be specifically targeted by a recombinant fragment ID1-DBL2X-ID2a (rVAR2) of the *Plasmodium falciparum* VAR2CSA protein (27, 28, 29). In a preliminary study, we found that rVAR2 expressed in *E. coli* BL21(DE3) could interact with not only CS-A but also *Dosidicus gigas* cartilage-derived CS-E (30) and porcine intestinal mucosa-derived Hep (Fig. S1), indicating that rVAR2 has the potential to recognize other GAG-based epitopes. In this study, we used rVAR2 as a template and cancer cell-derived GAGs as targets to identify new probes that specifically recognize GAG-based epitopes, by performing random mutation and using phage display technology. A novel probe, VAR2HP, with high selectivity for Hep was obtained and shown to target Hep-like epitopes with unique structural features in various cancer cells. This study provides strategy for development and identification of novel GAG-based epitopes and their specific probes.

## Results

### Screening of a probe VAR2HP binding to GAGs from tumor cells

**Figure 1:**
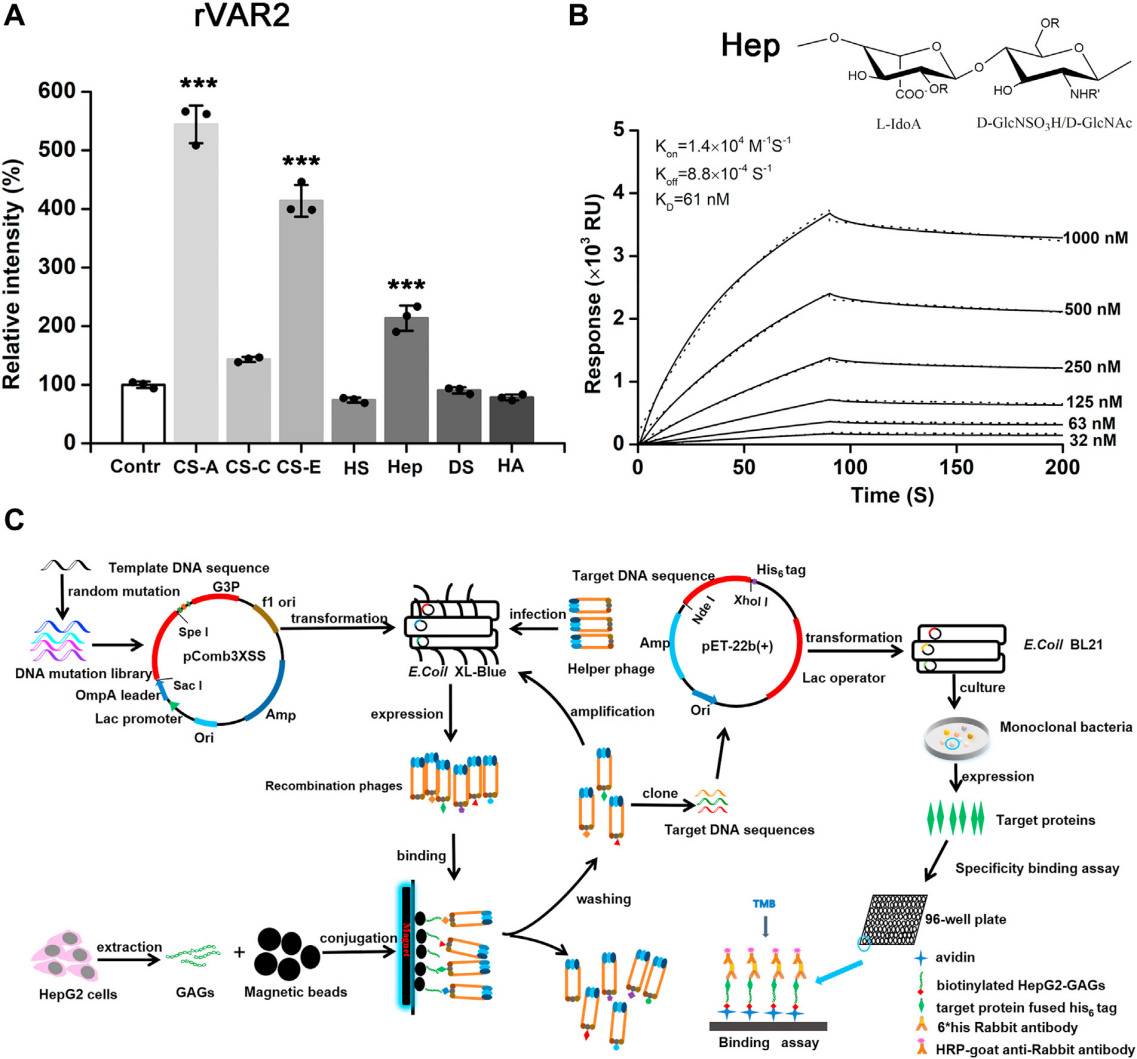
**Screening of a probe VAR2HP.***A*, binding capacity of rVAR2 to various GAGs were analyzed by binding assay. The data are shown as the ratio of the absorbance value of the test groups to that of the control group (contr) without immobilized GAGs; all values were subtracted from the value obtained for the blank, which did not contain recombinant protein. *p*-values are compared with control group, ∗∗∗*p* < 0.001. *B*, sensorgrams show the binding of rVAR2 at the indicated concentration (32–1000 nM) to Hep immobilized on a sensor chip in SPR analysis using a BIAcore T2000. *RU*, resonance units. K_D_ value was calculated by K_off_/K_on_. *Black line* represents data, *dot line* represents fitted curves by a 1:1 binding model. *R*: H/SO_3_^−^, *R’*: SO_3_^−^/Ac in (*B*). *C*, schematic diagram showing the phage display technology used in this study. CS, chondroitin sulfate; DS, dermatan sulfate; GAGs, glycosaminoglycans; HA, hyaluronan; Hep, heparin; HS, heparan sulfate; SPR, surface plasmon resonance.

The gene of ID1-ID2a (VAR2) domain was codon-optimized and synthesized based on the previously reported protein sequence (29), cloned into pET-22b (+) vector, and then expressed by *E. coli* BL21 (DE3). The recombinant VAR2 (rVAR2) was purified to >95% purity by nickel affinity chromatography coupled with gel filtration (Fig. S2*A*) and tested for its binding ability to various GAGs using a binding assay. The results showed that the obtained rVAR2 could not only strongly interact with CS-A but also significantly interact with CS-E and Hep (Figs. 1*A* and S2*C*), and especially a surface plasmon resonance binding analysis showed that rVAR2 bond to Hep with a high affinity (K_D_ ∼ 61 nM) (Fig. 1*B*). Notably, the interaction of rVAR2 with Hep and CS-E has not been investigated in previous studies, which may limit knowledge about the selectivity of rVAR2 binding to GAGs. The binding of rVAR2 to CS-E may be due to the high proportion (>80%) of 4-*O*-sulfated GalNAc residues in CS-E (Table S1). In contrast, the possible reason for the binding of rVAR2 to Hep is unclear, but previous studies have shown that rVAR2 binds to CS-A through a positively charged groove (31, 32), which may also involve in the interaction with the highly negatively charged Hep. Anyway, this finding provides us with the opportunity and possibility to use it as a template to screen Hep-selective probes. To screen novel probes for the GAG-based epitopes of tumors, rVAR2 was exploited as a starting template, and then, random mutation was combined with phage display technology to screen probes that directly target cancer cell GAGs. As shown in Figure 1*C*, HCC HepG2 cell-derived GAGs (HepG2-GAGs) containing 95.88% of Hep/HS and 4.12% of CS/DS (Fig. S3 and Table S2) were biotinylated, preincubated with avidin, and then immobilized on carboxyl-modified magnetic beads. Then, a library of rVAR2 mutants was constructed *via* random mutations and expressed in a phage display. After multiple rounds of biopanning with the HepG2-GAGs-modified magnetic beads, recombinant phages carrying the targeting protein were retained. The genes encoding the target proteins were cloned into *E. coli*, the monoclonal bacteria were selected, and the selectivity of the recombinant proteins was analyzed by binding assay after heterologous induced expression. Finally, six clones (GAG-1 to GAG-6) with significant binding capacity to HepG2-GAGs were obtained (Fig. S2*B*), among which GAG-4 with the highest binding ability to HepG2-GAGs, named VAR2HP, was selected for further study.

### Sequence analysis and recombinant expression of VAR2HP

**Figure 2:**
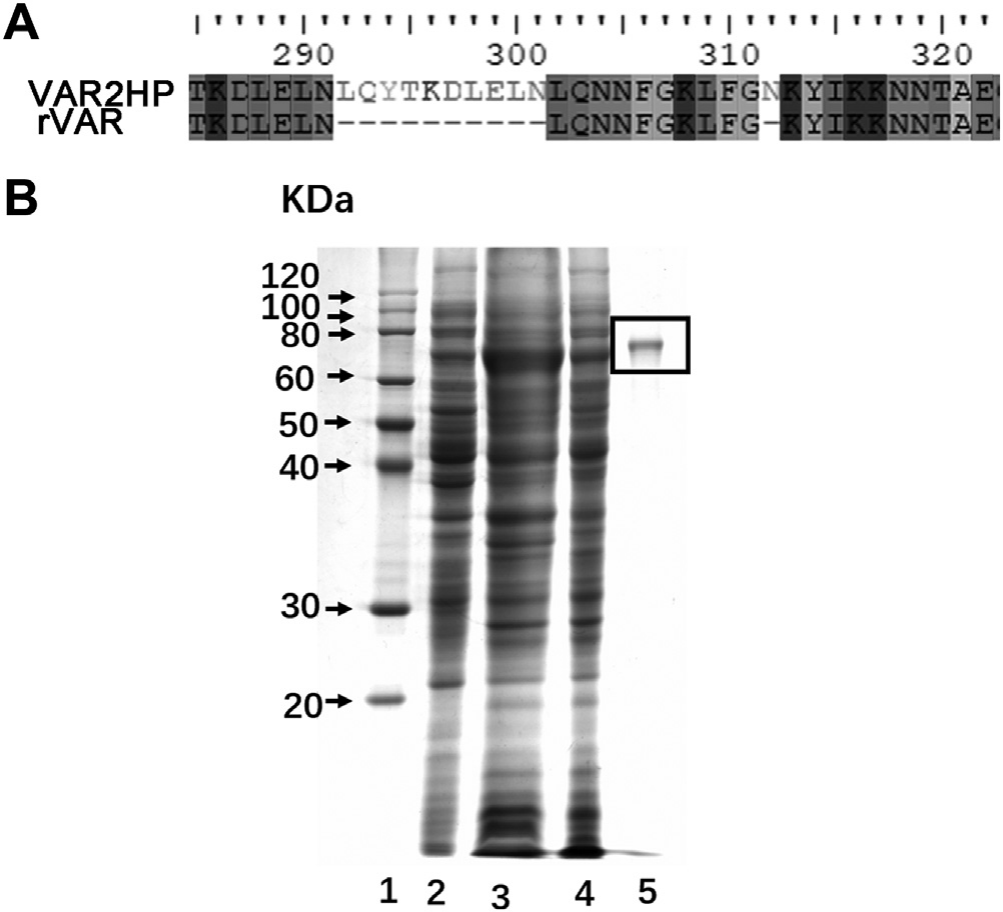
**Sequence and purification of VAR2HP.***A*, schematic showing the changes in the sequences between VAR2HP and ID1-DBL2X-ID2a (rVAR2). *B*, heterologous expression and purification of VAR2HP. Recombinant VAR2HP expressed in *E. coli* BL21 (DE3) was purified by nickel affinity chromatography followed by gel filtration and analyzed by SDS-PAGE under reducing conditions. Lane 1, Marker (ProteinRuler II, TransGen Biotech); Lane 2, uninduced cell lysate; Lane 3, induced cell lysate; Lane 4, supernatant fluid of induced cell lysate; Lane 5, purified proteins (*black box*).

After sequencing, the var2hp gene was shown to consist of 1953 bp with a GC content of 51.56% and to encode a protein containing 651 amino acids with an isoelectric point (PI) of 8.10, and a calculated molecular weight of 73.07 kDa using the peptide mass tool on the ExPASy server of the Swiss Institute of Bioinformatics. Compared with the sequence of rVAR2, VAR2HP has eleven extra amino acids, including a fragment L^292^C^293^Y^294^T^295^D^296^K^297^L^298^E^299^L^300^ N^301^ and an N^312^ residue (Fig. 2*A*). Then, the above obtained *E. coli* BL21 (DE3) cells harboring pET22b-var2hp were expanded and induced with isopropyl 1-thio-β-D-galactopyranoside, and after sonication and centrifugation, the VAR2HP protein was purified from the resulting lysis supernatant by a nickel affinity chromatography. SDS-PAGE showed VAR2HP was purified as a single band at approximately 70 KDa (Fig. 2*B*), which was consistent with the theoretical calculated value. The VAR2HP protein was preserved in PBS buffer for the following study.

### Selectivity of VAR2HP binding to GAGs

**Figure 3:**
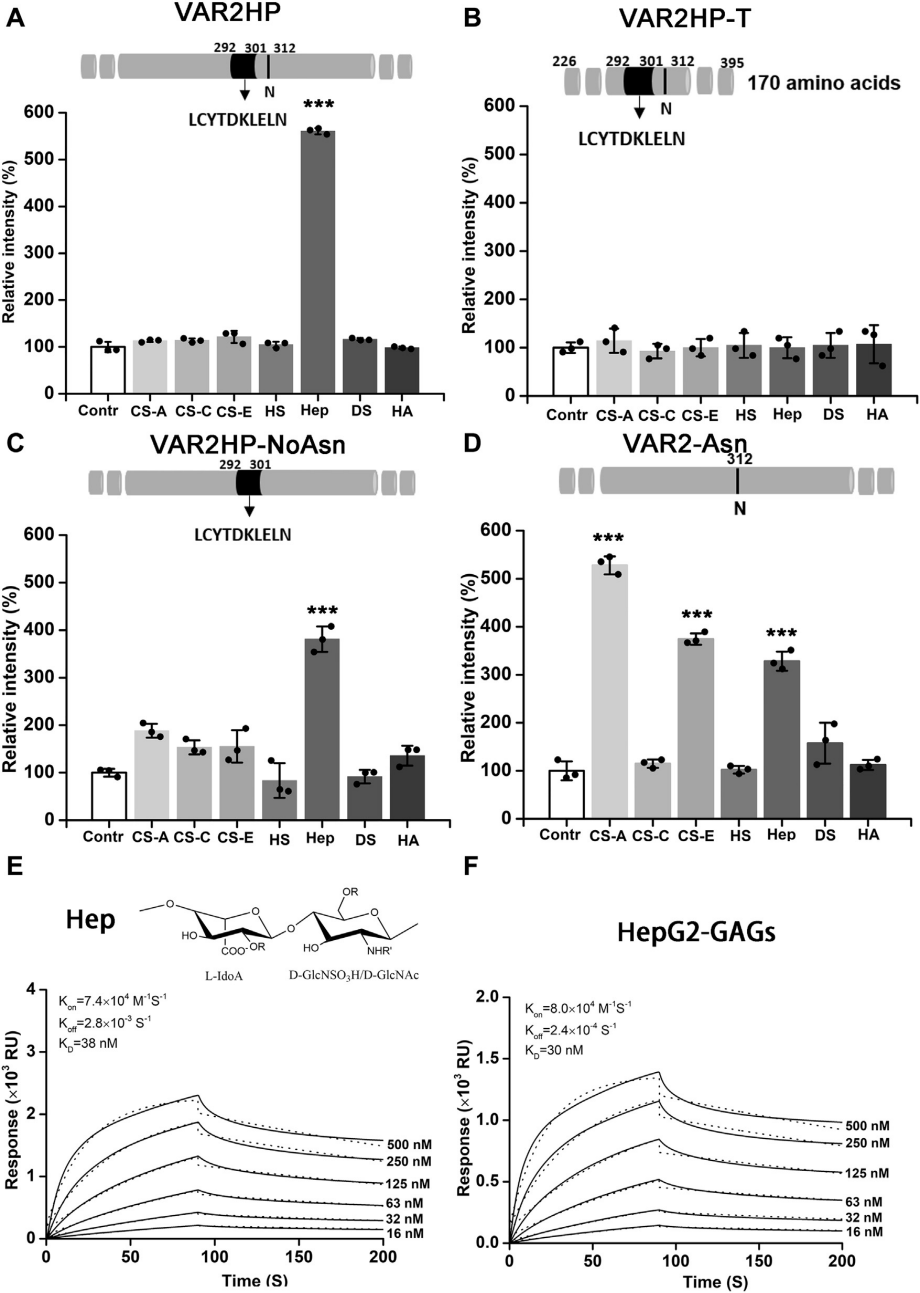
**Binding analysis of recombinant proteins.** Binding capacities of VAR2HP (*A*), VAR2HP-T (*B*), VAR2HP-NoAsn (*C*), and VAR2-Asn (*D*) to various GAGs were analyzed by binding assay. The data in each figure are shown as the ratio of the absorbance value of the test groups to that of the control group (Contr) without immobilizing biotinylated GAGs, in which all values were subtracted the blank value without recombinant protein (*p*-values in all cases are compared with control group, ∗∗∗*p* < 0.001). In the schematic illustration of mutants, *gray fragments* represent the composition of rVAR2, and *black fragments* represent the inserted amino acids in VAR2HP. Sensorgrams show the binding of VAR2HP to Hep (*E*) or HepG2-GAGs (*F*). *RU*, resonance units. K_D_ value was calculated by K_off_/K_on_. *Black lines* represent data, *dot lines* represent fitted curves by a 1:1 binding model. *R*: H/SO_3_^−^, *R’*: SO_3_^−^/Ac in (*E*). GAGs, glycosaminoglycans.

To investigate the selectivity of VAR2HP, binding assay was performed to analyze the binding capacity of VAR2HP to various GAGs (Table S1). Under optimal conditions (Fig. S4), the results showed that VAR2HP significantly bound to Hep but not to the other GAGs (Fig. 3*A*) and to Hep at a level comparable to that of rVAR2 to CS-A (Fig. S2*C*). Additionally, no obvious binding to CS-A or HS was detected with increasing concentrations of VAR2HP (Fig. S5), suggesting that VAR2HP, unlike rVAR2, is a Hep-selective probe.

To further investigate the roles of two mutation sites (L^292^C^293^Y^294^T^295^D^296^K^297^L^298^E^299^L^300^ N^301^) and N^312^ in VAR2HP binding to Hep, three mutants were recombinantly constructed and expressed using specifically designed primers (Table S3): VAR2HP-T, which was truncated VAR2HP (Glu^226^ to Asn^395^) containing the two mutation sites; VAR2HP-noAsn, which was a mutant of VAR2HP with N^312^ deleted; and VAR2-Asn, which was a mutant of VAR2 with an inserted Asn in the position corresponding to N^312^ of VAR2HP (Figs. 3, *B*–*D* and S2*C*). Binding assays showed that VAR2HP-T bound to no GAGs (Fig. 3*B*), indicating that the inserted fragment L^292^C^293^Y^294^T^295^D^296^K^297^L^298^E^299^L^300^ N^301^ and residue N^312^ together do not determine the binding capacity of VAR2HP to Hep. On the other hand, compared with VAR2HP, the mutant VAR2HP-NoAsn showed decreased binding to Hep and weak but significant binding to other GAGs (Fig. 3*C*), suggesting that the inserted N^312^ residue plays a key role in the selectivity of VAR2HP to Hep. However, the assay showing the binding of VAR2-Asn to GAGs indicated that the insertion of Asn^312^ alone did not meaningfully change the GAG-binding features of rVAR2, although a slight increase in binding activity to DS was observed (Fig. 3*D*). Taken together, these results suggested that the insertions of both fragment L^292^C^293^Y^294^T^295^D^296^K^297^L^298^E^299^L^300^ N^301^ and residue N^312^ resulted in a dramatic change in template rVAR2 GAG-binding properties and endowed the mutant VAR2HP with strict selectivity for Hep. Surface plasmon resonance showed that VAR2HP strongly interacts with Hep (K_D_-value of 38 nM), although the affinity is slightly lower than that (K_D_ ∼ 30 nM) with HepG2-GAGs (Fig. 3, *E* and *F*).

### Structural characteristics of epitopes recognized by VAR2HP

**Figure 4:**
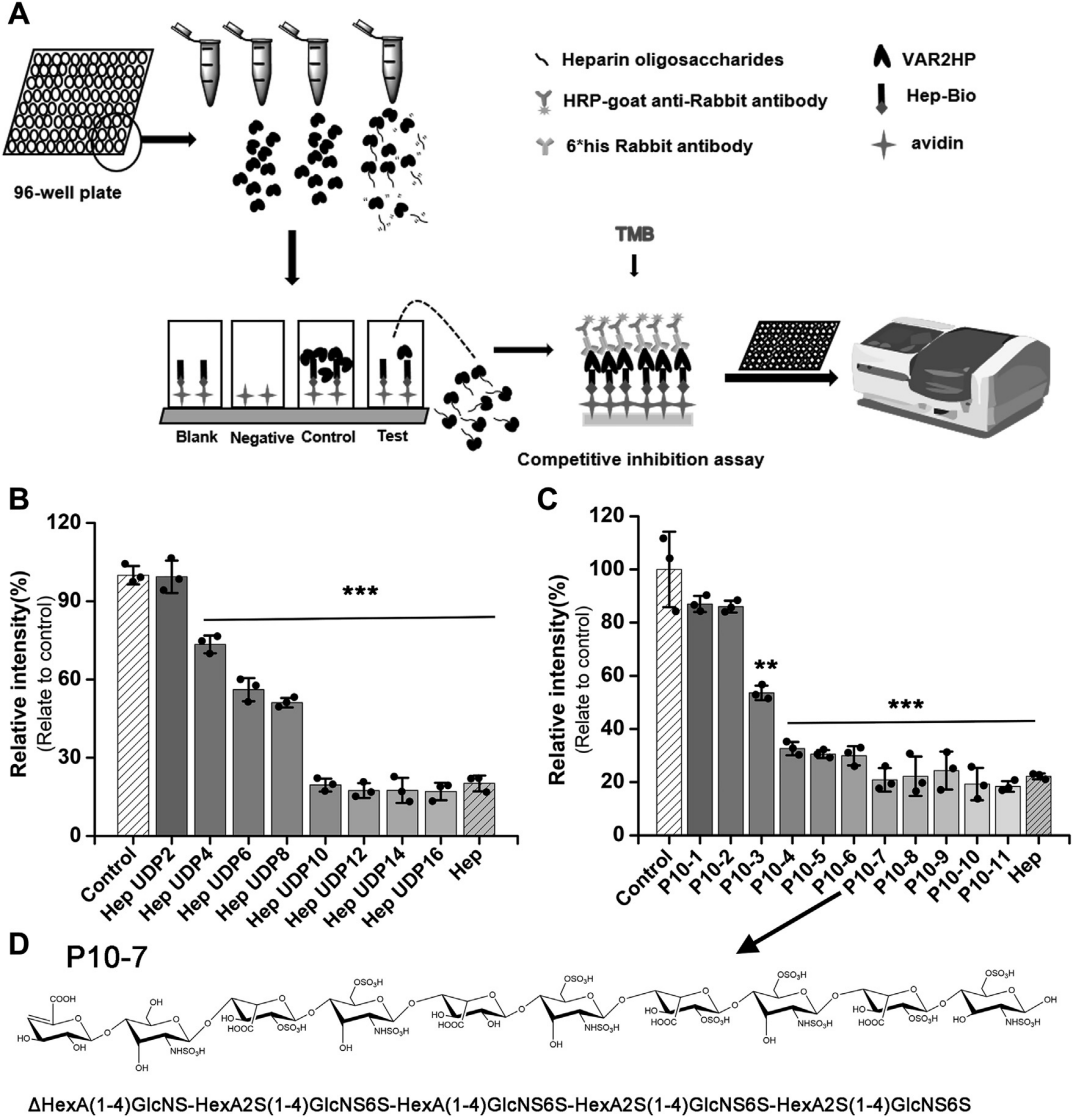
**The binding of VAR2HP to Hep oligosaccharides.***A*, schematic diagram showing the results of the competitive inhibition assays. *B*, competitive inhibition of VAR2HP to immobilized Hep by size-defined Hep oligosaccharides. *C*, competitive inhibition of VAR2HP to immobilized Hep by decasaccharide subfractions (∗∗*p*< 0.01, ∗∗∗*p* < 0.001, compared to control group without the addition of oligosaccharides as inhibitors). *D*, preliminarily obtained sequence of P10-7.

Although VAR2HP was selected by using HepG2-GAGs as targets, it is usually very difficult to prepare enough GAGs from tumor cells for detailed structural analyses. Therefore, as an alternative, commercially available Hep was used to investigate the structural characteristics of epitopes recognized by VAR2HP. First, a series of unsaturated even-numbered oligosaccharides from disaccharides to hexadecasaccharides (UDP2 to UDP16) with various disaccharide compositions (Table S4 and Fig. S6) were prepared through the partial digestion of Hep with heparinase II (Hepase II) (33) and used as inhibitors to investigate the Hep motif required for binding by VAR2HP through a competitive inhibition assay under the optimized inhibitor amount of 0.1 μg (Figs. 4*A* and S7). The results showed that the oligosaccharides exhibited increased activity with increasing molecular size. Notably, the inhibitory effects of the UDP4 to UDP8 oligosaccharides were much weaker than those of the decasaccharide (UDP10) and larger oligosaccharides, which showed effects similar to those of polysaccharide Hep (Fig. 4*B*). These results showed that decasaccharide is the essential motif with binding affinity for VAR2HP, similar to Hep polysaccharide.

**Figure 5:**
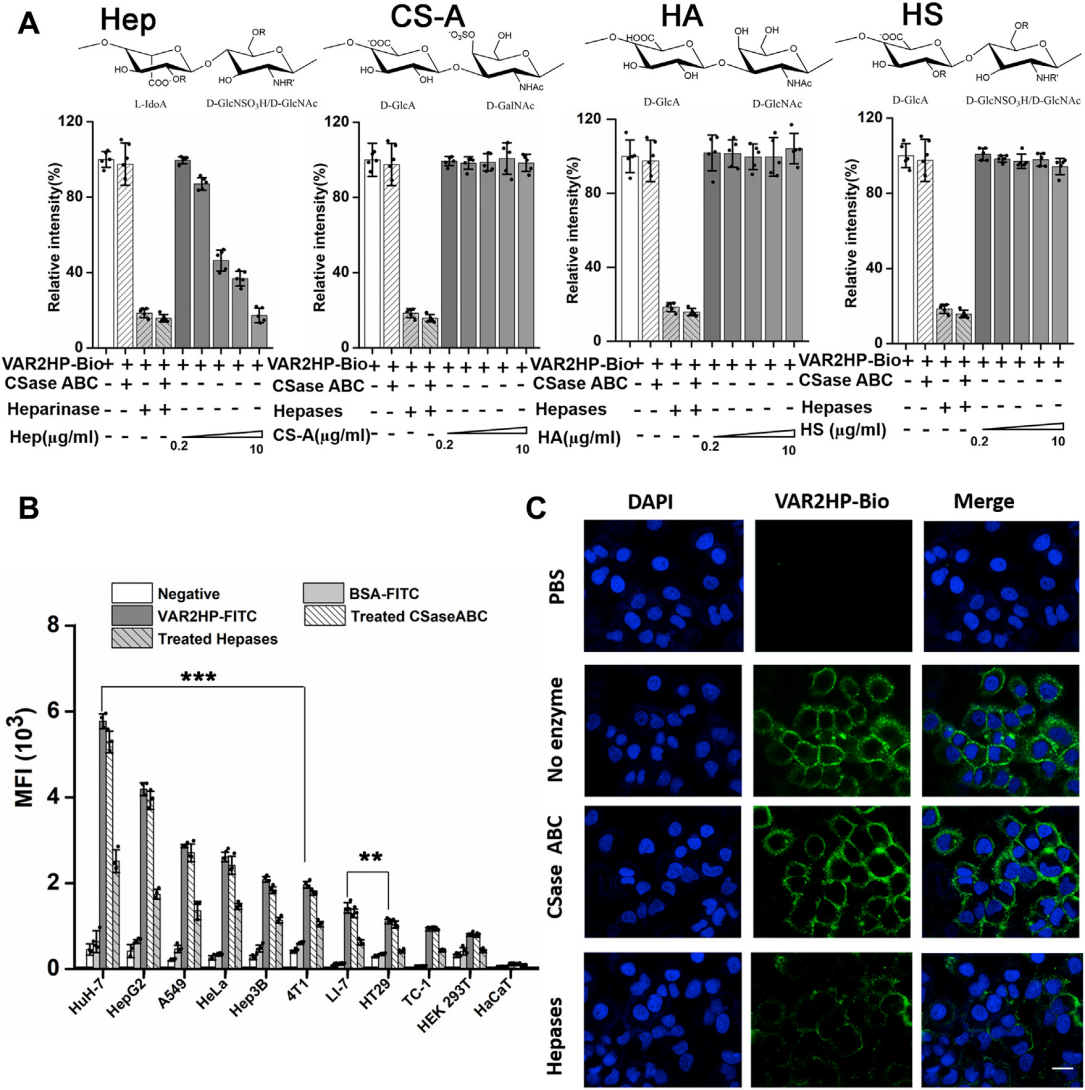
**Selective binding and localization of VAR2HP on cells.***A*, binding assay of VAR2HP-Bio to HuH-7 cells treated without or with Hepases or/and CSase ABC or in the presence of Hep, CS-A, HA, or HS at different concentrations (0.2, 2.5 5, 7.5, and 10 μg/ml, respectively). *R*: H/SO_3_^−^, *R’*: SO_3_^−^/Ac. *B*, flow cytometry assay of VAR2HP-FITC binding to various cells treated without or with Hepases or CSase ABC (∗∗*p*< 0.01, ∗∗∗*p* < 0.001, the binding of VAR2HP-FITC to tumor cells was compared to that to TC-1 cells with the highest binding among three normal cell lines). *C*, cells stained with VAR2HP-Bio. HuH-7 cells treated without or with Hepases or CSase ABC were also stained with VAR2HP-Bio (*green*) and DAPI (*blue*). The scale bar represents 20 μm. CS, chondroitin sulfate; HA, hyaluronan; Hep, heparin; HS, heparan sulfate.

The sulfation pattern of the Hep chain also plays a key role in Hep interaction with specific target proteins (34, 35, 36). To further characterize the structure of the Hep motif recognized by VAR2HP, the Hep UDP10 was subfractionated by anion-exchange HPLC (Fig. S8), and the main subfractions (P10-1 to P10-11) were pooled for the competitive inhibition assay. From the general trend, the inhibitory activity increased with the increasing anion strength of decasaccharides eluted with higher concentrations of salt (Figs. 4*C* and S8), indicating the importance of oligosaccharide charge intensity in the interaction. In detail, the inhibitory activity in subfractions P10-3, P10-4, and P10-7 showed stepwise increases (Fig. 5*C*), indicating the subtle influence of oligosaccharide structure on the interaction. Moreover, in contrast to the decasaccharides P10-1 to P10-3 with low activity levels, all the highly active decasaccharides (P10-4 to P10-11), contained at least three trisulfated disaccharides on average (Table S5). Interestingly, the inhibitory activity of subfractions P10-4 to P10-11 were not always positively correlated with their charge intensity, for example, the charge intensity of oligosaccharides from P7 to P11 increased obviously but their inhibitory capacity did not increase significantly (Fig. 4*C* and Table S5), indicating that the binding capacity of these Hep oligosaccharides to VAR2HP depends not only on their charge intensity but also on their structure, such as their sequences/sulfation patterns. To further investigate the structural characteristics of the Hep motifs recognized by VAR2HP, subfraction P10-7, which showed inhibitory activity similar to that of the Hep polysaccharide (Fig. 4*C*), was repurified through gel filtration, and the main fraction was preliminarily sequenced with an enzymatic method that we recently developed (33). The results showed that it contained three types of disaccharides: HexA(1–4)GlcNS, HexA(1–4)GlcNS6S, and HexA2S(1–4)GlcNS6S at a ratio of 1:1:3 (Fig. S9). In terms of sequence, these three trisulfated HexA2S(1–4)GlcNS6S disaccharides were discontinuous in the oligosaccharide chain (Fig. 4*D*), which differed from the HS motif (GlcNS6S-IdoA2S)_3_ recognized by the scFv antibody NS4F5 (17).

### Hep-like epitopes on tumor cells detected by VAR2HP

To investigate whether the Hep-like epitopes recognized by VAR2HP are expressed on cells other than HepG2 cells, VAR2HP was first biotinylated (VAR2HP-Bio) as described previously (37). After verifying the selectivity of VAR2HP-Bio to Hep and optimizing the corresponding assay conditions (Fig. S10), the cell binding assay results showed that VAR2HP-Bio very strongly bound to human HCC HuH-7 cells and that the binding was mostly disrupted by treatment with Hepase I, II, and III (Hepases), but not by chondroitinase ABC (CSase ABC), and was dose dependently inhibited by Hep rather than other GAGs (Figs. 5*A* and S11, *D*–*F*). Similar results were observed in the binding assay of VAR2HP-Bio to HepG2 cells, human cervical adenocarcinoma HeLa cells and 4T1 murine mammary carcinoma cells (Fig. S11, *A*–*C*). These results indicated that other cancer cells can also express Hep-like epitopes recognized by VAR2HP.

To quantitatively analyze the expression of these Hep-like epitopes on various cells, fluorescein isothiocyanate (FITC)-labeled VAR2HP (VAR2HP-FITC), which had been shown to retain selective binding activity to Hep (although its emission wavelength was slightly redshifted compared to FITC (Fig. S12), was used as a probe to detect Hep-like epitopes on various living cancer and normal cells by flow cytometry. The results showed that the binding of VAR2HP-FITC was highly cell-type dependent and that it preferentially bound certain cancer cells, including human HCC HuH-7, HepG2, and Hep3B2.1--7 (Hep3B) cells, lung carcinoma A549 cells, and HeLa cells, as well as 4T1 cells (Fig. 5*B*). In contrast, normal HEK 293T cells, TC-1 cells, HaCaT cells, and human colon adenocarcinoma HT29 cells were very weakly stained by VAR2HP-FITC (Fig. 5*B*), suggesting that they likely express very low levels of VAR2HP-targeted epitopes compared with the aforementioned highly stained cancer cells. Unsurprisingly, the staining of various cells by VAR2HP-FITC was strongly inhibited by pretreatment with Hepases but not with CSase ABC (Fig. 5*B*), confirming the expression of Hep-like epitopes on various cells.

Based on the structural properties of the Hep epitopes targeted by VAR2HP, we speculate that the content of HexA2S(1–4)GlcNS6S in HS chains on the cell surface is one of key factors for the binding of VAR2HP. Disaccharide composition analysis showed that HS chains extracted from all test cells contained HexA2S(1–4)GlcNS6S residues (Figs. 5*B*, S3*A* and S13; Tables S2 and S6). Consistent with their highest VAR2HP-binding capacity among all the tested cells (Fig. 5*B*), HuH-7 cells have the highest percentage (12.34%) and content (208.43 pmol) of HexA2S(1–4)GlcNS6S in 1 mg of dry cells. In contrast, HCC Li-7 cells express much lower amount of HexA2S(1–4)GlcNS6S (30.69 pmol) than HCC HuH-7 cells (208.43 pmol) and HepG2 cells (173.90 pmol) (Tables S2 and S6), which may explain why Li-7 cells showed much lower VAR2HP-binding ability than HuH-7 and HepG2 cells. Similarly, the very low binding of VAR2HP to HT29 and HEK 293T cells should be due to the rare expression of HexA2S(1–4)GlcNAcS6S (8.68 pmol and 9.42 pmol, respectively) on these cells (Table S6 and Fig. 5*B*). Clearly, the amount of trisulfated HexA2S(1–4)GlcNAcS6S expressed on cells is one of important factors in determining the VAR2HP-binding capacity of cells. However, it should be noted that 4T1 cells express a lower content (28.23 pmol) and proportion (4.81%) of HexA2S(1–4)GlcNAcS6S than Li-7 cells (30.69 pmol, 7.14%) but show a higher VAR2HP-binding capacity, indicating that the content of HexA2S(1–4)GlcNAcS6S expressed on cells is not the only key factor for VAR2HP-binding. Undoubtedly, the most important thing is that these trisulfated disaccharides can efficiently form the VAR2HP-binding epitopes in the HS chains.

Finally, to investigate the localization of VAR2HP on cells, HuH-7 cells were stained with VAR2HP-Bio and visualized by confocal microscopy. The results showed that VAR2HP-Bio (green) clearly bound to the cell membrane, and the staining was eliminated by treatment with Hepases but not CSase ABC (Fig. 5*C*), further confirming that VAR2HP bound to HS/Hep but not to CS/DS chains on the cell surface.

**Figure 6:**
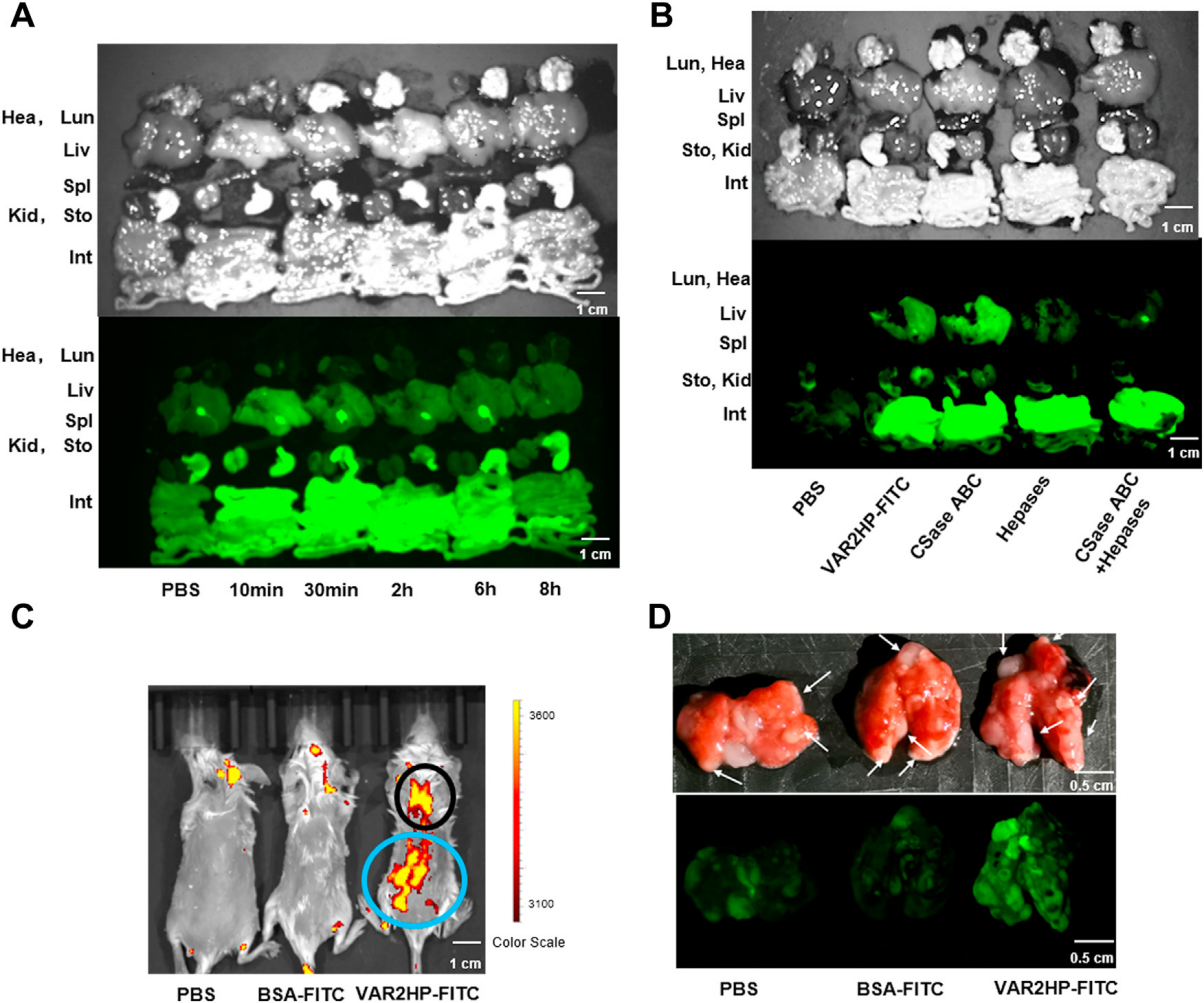
**The localization and selectivity analysis of VAR2HP *in vivo*.***A*, visualization of VAR2HP-targeted organs. *B*, effects of enzyme treatment on VAR2HP targeting of organs. *C*, *in vivo* image showing tumor-bearing mice. The lung with tumor nodules is marked with a *black circle*. *D*, fluorescence image showing the lungs with tumor nodules. *White arrows* indicate the tumor nodules.

### Selectivity of VAR2HP to tumor tissues *in vivo*

To further explore the possibility of application *in vivo*, the targeting of VAR2HP to organs *in vivo* was first investigated by injecting VAR2HP-FITC into mice through the tail vein. VAR2HP-FITC mainly targeted the intestine, stomach, and liver and could remain in these organs for at least 6 h (Fig. 6*A*), indicating that VAR2HP has good biocompatibility, which led to delayed immune clearance, similar to its template protein rVAR2 (27). Previous studies demonstrated that GAG lyases could be used to degrade GAGs *in vivo* (38, 39, 40, 41), and thus Hepases and CSase ABC were preinjected into mice through the tail vein to prove the binding of VAR2HP to organs *via* Hep-like epitopes. As shown in Figure 6*B*, VAR2HP-FITC targeted to the liver, kidney, and stomach was almost eliminated by pretreatment with Hepases but not CSase ABC, confirming that the Hep/HS chains expressed in these organs played a key role in VAR2HP targeting. Furthermore, the disaccharide composition analysis showed that HS/Hep extracted from the liver, kidney, and stomach contained relatively more trisulfated HexA2S(1–4)GlcNS6S disaccharides than the spleen, lung, or heart (Table S7 and Fig. S14), which may explain why VAR2HP preferentially targeted the stomach, liver, and kidney. Notably, although the proportion of HexA2S(1–4)GlcNS6S in HS/Hep from the intestine was even lower than that in the spleen (Table S7 and Fig. S14), the content of HexA2S(1–4)GlcNS6S in the intestine was the highest among all organs, indicating that the intestine may contain more Hep-like epitopes on which VAR2HP-FITC could bind.

To study whether VAR2HP targets tumors *in vivo*, lung tumor-bearing Balb/c mouse model was constructed with 4T1 cells, and then, they were injected with VAR2HP-FITC through the tail vein. *In vivo* images showed that the VAR2HP-FITC group had the highest fluorescence intensity in the liver and intestine area (blue circle) and lung area (black circle) (Fig. 6*C*), which can likely be attributed to the targeting of VAR2HP-FITC to tumors in the lungs. To confirm this speculation, the lungs were evaluated by fluorescence imaging analysis, and tumor nodules (white arrow) showed much higher fluorescence intensity than the surrounding tissue (Fig. 6*D*). These results suggested that VAR2HP can be a promising carrier for selectively delivering drugs to tumors overexpressing Hep-like epitopes *in vivo*.

## Discussion

GAGs aberrantly expressed by tumor cells have the potential to be epitopes for the diagnosis and targeted therapy of cancers, and several scFv antibodies against GAGs with unique structures, such as HS20 (18) and GD3G7 (23), have been shown to be promising probes for targeting such tumor-associated epitopes. However, these scFv antibodies were selected from some semisynthetic scFv libraries by using phage display technology, which were not specifically established for screening probes against GAG-type epitopes on tumor cells (42, 43, 44). In this study, we have developed a method to directly screen probes to selectively target tumor GAGs by combining random mutation of GAG-binding protein (*e.g.*, rVAR2), phage display, and screening using tumor-derived GAGs. The protein rVAR2, which is the ID1-DBL2X-ID2a region of VAR2CSA that mediates the binding of *P. falciparum* infected erythrocytes to placental syncytiotrophoblasts (45), has been shown to have high affinity binding to CS-A-like epitopes expressed in the placenta and various cancers. In this study, however, we found that rVAR2 can bind to multiple GAGs including CS-E and Hep in addition to CS-A and thus has the potential to be used as the parent template to screen and optimize GAG-specific probes through mutation. In addition, the separation technology using magnetic beads modified with HepG2-GAGs could improve the screening efficiency of probes due to its semiautomated and high-through characteristics (46). As expected, the identification of Hep-selective VAR2HP demonstrates the practicability and effectiveness of the method in screening and selectivity optimization of probes for GAGs. Technically, this study provides a strategy for the purposeful screening and optimization of GAG-specific probes based on the selection of suitable GAG targets and GAG-binding templates including scFv antibodies against GAGs. Compared to its parental template rVAR2, which binds to CS-A, CS-E, and Hep, VAR2HP exhibits high selectivity for Hep (Fig. S2*C*). Based on their sequences, VAR2HP is a mutant of rVAR2 with insertion of fragment L^292^C^293^Y^294^T^295^D^296^K^297^L^298^E^299^L^300^N^301^ and residue N^312^. Binding assays with various mutants of VAR2HP have shown that both the fragment L^292^C^293^Y^294^T^295^D^296^K^297^L^298^E^299^L^300^N^301^ and residue N^312^ play a key role but not the only role in the selectivity of VAR2HP for Hep (Fig. 3, *B*–*D*). Previous studies have shown scFv antibodies interact with Hep/HS occurs *via* some basic residue-containing sequences, such as XBBXBX, XBBBXXBX, and XBBBXXBBBXXBBX, where ‘B’ is a basic residue and ‘X’ is a random amino acid residue (47). This kind of interaction based on electrostatic attraction is easily disrupted by various environmental factors and is usually not sufficiently specific, especially in complex environments, such as cells and tissues. According to the primary structural sequence, VAR2HP with a theoretical PI of 8.1 is not highly positively charged at physiological pH like some other GAG-binding proteins (48) nor does it possess highly positively charged fragments like the GAGs-binding regions of scFv antibodies (47), so the interaction of VAR2HP with Hep-like epitopes is not simply due to charge interaction but due to its unique tertiary structure, which may locally form a positively charged groove, as the case of VAR2 bound to CS-A (31, 32). Therefore, VAR2HP should be a stable probe for detecting and identifying the location of Hep-like epitopes *in vitro* and *in vivo*. Indeed, its applicability in complex biological environments was well demonstrated by the selective targeting of Hep-like epitopes on cells *in vitro* (Figs. 5, S3*A*, and S13) and in tissues *in vivo* (Fig. 6, *C* and *D*).

The structural characteristics of epitopes recognized by VAR2HP were investigated by using oligosaccharides prepared from commercial Hep instead of rare HS extracted from cancer cells. The results have shown that the structure with similar affinity for VAR2HP as Hep polysaccharide is decasaccharides containing at least three HexA2S(1–4)GlcNS6S disaccharides (Figs. 4, *C* and *D* and S9). Interestingly, the trisulfated HexA2S(1–4)GlcNS6S is also an essential structural element in the epitopes of the scFv antibodies NS4F5 (17) and HS20 (19), which may be due to its high negative charge. However, the binding activity of Hep decasaccharides to VAR2HP does not always increase with their degree of sulfation (Fig. 4*C* and Table S5), indicating that the interaction with VAR2HP is attributed to both the charge intensity and structural features of epitopes. In general, the Hep-like epitopes recognized by VAR2HP are not a single structure but have some unique structural features in terms of size, disaccharide composition, and very possibly sequences that remain to be studied in the future. Cell staining showed that these Hep-like structures were preferentially overexpressed by various cancer cells (Figs. 5, S3*A* and S13). Similarly, some Hep-like epitopes were detected by the scFv antibodies NS4F5 (17) and HS20 (18) in ovarian cancer and HCC, respectively. These results from different studies suggest that these Hep-like structures could be associated with the malignancy of cells and could be used as potential tumor markers. However, the structural features of these epitopes may be unique for each probe that recognizes different tumor cells, and the structures recognized by VAR2HP may be overexpressed by a wider variety of cancer cells, making VAR2HP a potential broad-spectrum probe to target different tumors.

The VAR2HP-binding capacity of various cancer cells, such as HuH-7, HepG2, 4T1, HeLa *et al.*, could be specifically inhibited by treatment with Hepases or coincubation with Hep (Figs. 5*A* and S11), and a disaccharide composition assay of HS extracted from these cells showed that the content of trisulfated HexA2S(1–4)GlcNS6S was related to this capacity (Tables S2 and S6, Figs. S13, and S3*A*), which suggests that Hep-like HS on the cell surface is involved in the interaction with VAR2HP. However, it is worth noting that the content (6.56%) of HexA2S(1–4)GlcNS6S in HS from HepG2 cells is much lower than that (65.34%) in Hep but the affinity (K_D_ ∼ 30 nM) of VAR2HP with HepG2-GAGs is higher than that (K_D_ ∼ 38 nM) with Hep (Tables S4 and S6, Fig. 3, *E* and *F*). These results indicate that the VAR2HP epitopes on cancer cells have some unique structural features compared with Hep, which needs to be further investigated. Among the cells used in this study, HCC cells such as HuH-7 and HepG2 were strongly stained *in vitro*, and mouse liver was targeted *in vivo* by VAR2HP, indicating that HCC cells and normal liver tissues are rich in the VAR2HP epitopes (Figs. 5*B* and 6*A*). As an exception, HCC Li-7 cells were very weakly stained by VAR2HP, which may be due to the overexpression of CSPG CD44 on their cell surface rather than HSPG glypican-3 like other HCC cells (49).

*In vivo* experiments further proved that it had good biocompatibility and could remain in the complex environment of the body for more than 6 h (Fig. 6*A*). These properties of VAR2HP are very similar to the case of its parental template rVAR2 targeting cells with CS-A-like epitopes (27). Compared with rVAR2 binding to multiple GAGs including CS-A, CS-E, and Hep, as shown in this study (Figs. 1*A* and S2*C*), the high selectivity of VAR2HP for Hep-like epitopes may limit its binding to more cell types but could increase its selectivity for cancer cells to reduce damage to normal cells in targeted therapy. *In vivo*, VAR2HP can target the liver, stomach, kidney, and intestine due to the relatively high content of HexA2S(1–4)GlcNS6S in their HS/Hep (Figs. 6*A* and S14; Table S7). However, differing from other organs, the intestine targeting seems to be weakly affected by treatment with Hepases (Fig. 6*B*), indicating that some other factors may be involved in the binding or interfere with enzyme action in the intestine. Obviously, this organ-targeting issue will raise concerns that tumor-targeted therapy with VAR2HP may cause harm to these organs. In fact, since tumor antigens are always more or less expressed in normal tissues and organs, the side effects of tumor-targeted therapy are inevitable, but it can promote anticancer drug accumulation in tumor at reduced doses, thereby minimizing or avoiding undesired side effects (3, 50). *In vivo* imaging showed that VAR2HP-FITC was located in 4T1-tumor tissue rather than normal tissue in the lung (Fig. 6, *C* and *D*), suggesting that VAR2HP has a greater affinity for the Hep-like epitopes overexpressed in tumors and has potential for targeted therapy.

In conclusion, this study provides a common platform for purposefully screening and optimizing GAG-specific probes by choosing appropriate GAG targets and GAG-binding templates including scFv antibodies against GAGs, which are urgently needed for not only tumor targeted therapy but also other structural and functional studies of various GAGs.

## Experimental procedures

### Materials

The DNA sequence of ID1-DBL2X-ID2a domains of VAR2CSA was downloaded from National Center for Biotechnology Information (NCBI) database as published (29), codon-optimized, and synthesized by GENEWIZ, Inc. Taq DNA polymerases, PrimerStar Max DNA polymerases, and restriction endonucleases XhoI, SpeI, SacI, and NdeI were purchased from Takara, Inc. Mut Express II Fast Mutagenesis Kit V2, 5 min TA/Blunt-Zero Clone Kit, *E. coli*. DH5α cells, and *E. coli*. BL21(DE3) cells were purchased from Vazyme Biotech, Co Ltd. ProteinRuler II was purchased from TransGen Biotech, Inc. The expression plasmid pET-22b (+) was purchased from Invitrogen. The plasmid vector pComb3XSS was obtained from the Barbas Laboratory, TSRI. The VCSM13 helper phage and host bacteria *E. coli*. XL-blue cells were purchased from Bio-view Shine, Inc. CSase ABC (EC 4.2.2.4), Hepases I (EC 4.2.2.7), II (EC 4.2.2.-), III (EC 4.2.2.8), hyaluronan from *streptococcus equi*, CS-A from bovine trachea, CS-C from shark cartilage, avidin from egg whites, and FITC were purchased from Sigma-Aldrich, Inc. DS from porcine skin were obtained from Seikagaku Crop. *D. gigas* cartilage-derived CS-E was prepared in our laboratory (30). Porcine intestinal mucosa-derived Hep and low sulfated HS were obtained from Tiandong Pharma. The disaccharide composition of all GAGs is shown in Table S1. Size-defined Hep oligosaccharides were prepared by the digestion of Hep polysaccharides with Hepase II followed by size-exclusion chromatography on a Superdex Peptide 10/300 GL column in our laboratory (33). EZ-Link Biotin-LC-Hydrazide was obtained from Thermo Fisher Scientific. Horseradish peroxidase (HRP)-conjugated goat anti-Rabbit (IgG) secondary antibody, 6∗his Rabbit polyclonal antibody, and HRP-conjugated Streptavidin were obtained from Proteintech Group, Inc. Recombinant streptavidin protein-FITC was purchased from Abcam. The other chemicals and reagents were highest quality available.

### Cell lines and animals

HT29, 4T1, A549 cells were incubated in RPMI 1640 supplemented with 10% fetal bovine serum (FBS). HEK 293T, TC-1, HaCaT, HuH-7, HepG2, and HeLa cells were cultured in Dulbecco’s modified eagle medium supplemented with 10% FBS. Hep3B2.1-7 (Hep3B) cells were incubated in MEM supplemented with 10% FBS, 1% 100 mM L-Glutamine, 1% nonessential amino acid, and 1% sodium pyruvate. Li-7 cells were incubated in RPMI 1640 supplemented with 10% FBS, 1% 100 mM L-Glutamine, and 1% sodium pyruvate. Cell culture mediums were purchased from Sparkjade Scientific Instruments Co, Ltd. The Balb/c mice weighting 18.0 to 20.0 g were raised in comfortable environment, fed standard food, and allowed to drink freely. All animal experiments were approved by the Animal Care and Use Committee of Shandong University (Document number SYXK (LU) 2019005).

### Preparation of HepG2-derived GAGs-modified magnetic beads

HepG2-GAGs were extracted from HepG2 cells as described under “Extraction of GAGs from cells and tissues” below and further purified by anion-exchange chromatography as reported previously (21, 51). Briefly, crude HepG2-GAGs was loaded on a DEAE-Sephadex column (2 ml) pre-equilibrated with 50 mM phosphate buffer containing 0.2 M NaCl and eluted stepwise with 0.2, 0.5, and 2 M NaCl in 50 mM phosphate buffer. After checking with a carbazole reaction (52), the main GAG-containing fraction eluted by 2 M NaCl was desalted by Amicon Ultra-4 3K unit (Millipore) and lyophilized for further use. The content and purity (>80%) of the purified HepG2-GAGs was determined by a carbazole reaction (52). To immobilize HepG2-derived GAGs (HepG2-GAGs) on magnetic beads, HepG2-GAGs (5 mg) were biotinylated (HepG2-GAGs-Bio) with EZ-Link Biotin-LC-Hydrazide as reported previously (30) and incubated with avidin at molar radio 4:1 for 20 min at room temperature in PBS for preventing the biotin recognition site from being occupied by magnetic beads. Then, the compound of avidin and HepG2-GAG-Bio (HepG2-GAGs-Bio-avidin) was conjugated with FEO magnetic beads (Baseline) containing about 2.5 μmol carboxylic group using N-hydroxysuccinimide and 1-(3-dimethylaminopropyl-3-ethylcarbodiimide hydro according to the manufacturer’s instructions (Thermo Scientific). The magnetic beads were adsorbed on magnetic screening plate, and the supernatant was extracted to detect unbound protein and GAG by a BCA protein assay kit (CWBIO) and carbazole reaction method (52), respectively. The result showed that more than 90% HepG2-GAGs-Bio-avidin was bound to the surface of magnetic beads. The unbound carboxyl sites on the surface of magnetic beads were blocked by 0.5 M ethanolamine (pH 8.3), and the HepG2-GAGs-magnetic beads were stored in PBS at 4 °C for further study.

### Screening of HepG2-GAG binders by phage display technology

To build the phage mutation library, the plasmid mutation library of VAR2CSA ID1-DBL2X-ID2a with 6∗his tag at C terminal was amplified by primer pairs and low-fidelity Taq polymerase (Vazyme Biotech). The primer pairs (F’: 5′-GAGCTCCATATGAATTACATCAAAG-3, R’: 5′-ACTAGTGTGGTGGTGGTGGTGGTGCTCGAGATCCAGTTTGCTGC-3′, the underline in primers were restriction endonuclease recognition sites) shown in Table S3 were synthesized by Sangon Biotech. In order to improve mutate rate, bivalent manganese was added in PCR reaction system, the annealing temperature was decreased, and the PCR production was exposed to ultraviolet light 30 min (53). Phage display technology was carried out according to previously published (54). Briefly, Gel-recovered PCR products were cloned into pComb3XSS (pComb3XSS-mutVAR2) and transformed into *E. coli* XL-Blue cells. Phage helper VCSM13 infected *E. coli* XL-Blue cells to express recombinant plasmid which packaged of the pComb3XSS-mutVAR2 DNA and assembled of VCSM13 phage particles, yielding a library of infectious phages displaying the variant binding proteins. The library was added to the HepG2-GAGs-modified magnetic beads. The bound phages were eluted by PBS supplemented with 0.15 mM NaCl and then were used to produce more phages by re-infected *E. coli* XL-Blue cells with addition of helper phage VCSM13. The phages library was subjected to five rounds of above panning.

### Heterologous expression, selection, sequence analysis, and purification of the target proteins

The target sequences were amplified by primer pairs (F’: 5′-GAGCTCCATATGAATTACATCAAAG-3, R’: 5′-ACTAGTGTGGTGGTGGTGGTGGTGCTCGAGATCCAGTTTGCTGC-3′) using above enriched phages as templates. The sequences were constructed onto pET22b (+) vector (Novagen) and then transformed into *E. coli* BL21(DE3) cells. *E. coli* BL21(DE3) cells harboring different recombinant plasmids were selected by monoclonalization for following expression. Monoclonal bacteria were cultured in LB broth at 37 °C until cell density reached an *A*_600_ of 0.8 to 1.0 and then were added a final concentration of 50 μM isopropyl 1-thio-β-D-galactopyranoside to induce the expression of target protein. Cells were cultured at 16 °C for an additional 24 h and harvested by centrifugation at 8000*g* for 5 min. The cells were washed twice by ice-cold buffer A (50 mM Tris-HCl, 150 mM NaCl, pH 8.0), resuspended in buffer A, and disrupted by sonication with 60 repetitions per 5 s in ice-water bath environment. The cell lysate was centrifugated at 12,000*g* for 30 min to collect the supernatant. The capacities of the supernatant-contained recombinant proteins binding to GAGs were evaluated by “Binding assay” as following mentioned. The genes of proteins with high binding activity to GAGs were sequenced by Sangon Biotech, and compared by BioEdit, version 5.0, to search the differences from that of the template rVAR2. The PI of the proteins were predicted by ExPASy Compute pI/Mw tool.

Then, the lysate supernatants containing soluble target recombinant proteins with 6× his tagged at protein C terminal were loaded onto a column packed with pre-equilibrated nickel-Sepharose 6 Fast Flow (GE Healthcare) and eluted with a gradient concentration of imidazole in buffer A ranging from 0 to 500 mM. The fractions containing target protein were further purified by HPLC on a Superdex G-200 column using 25 mM Tris-Cl buffer containing 100 mM NaCl (pH 7.4) at monitoring 280 nm. Then the target protein was concentrated by Amicon Ultra 0.5-ml 10K unit (Millipore). The molecular weight of the purified protein was analyzed by 13.2% SDS-PAGE under reducing conditions, based on the method of Sambrook and Russell (55), and the concentration was detected by BCA protein Assay Kit (CWBIO).

### Binding assay

The binding assay was performed in 96-well plates according to previously published (37). After optimization of the experimental conditions including buffers (10 mM NaAc-HAc, pH 5.0–6.0; 10 mM Na_2_HPO_4_-NaH_2_PO_4_, pH 6.0–8.0; 10 mM Tris-HCl, pH 7.0–10.0), VAR2HP concentration (0–4 μg/ml), blocking agents (BSA, 1%–5%, skim milk, 1%–5%), and incubation time with target proteins (0–180 min), each well was coated with 0.4 μg avidin, blocked with 3% skim milk for 2 h, and then added 0.1 μg/well GAGs-Bio. After washed with PBS, the wells were added supernatant containing recombinant protein or purified recombinant protein (0.2 μg/well) in 3% skim milk for 2 h at room temperature and then rinsed three times with PBST (PBS supplemented with 0.05% tween-20) for removing nonspecific binding. Finally, the bound protein was detected by 6∗His Rabbit polyclone antibody, HRP-conjugated Goat anti-Rabbit (H + L), and 3,3′,5,5′-tetramethyl benzidine as a substrate. The wells were analyzed at 450 nm using microplate reader (BioTEK). Each experiment was performed in triplicates. The wells without GAG-Bio were used as the negative controls, and the wells with GAG-Bio but without adding recombinant proteins were used as the blanks.

To determine the binding capacity of VAR2HP to Hep oligosaccharides from di- to hexadecasaccharides (UDP2-UDP16) (33), a competitive inhibition assay was carried out by preincubating Hep polysaccharide or each size-defined Hep oligosaccharide fraction (0.1 μg, which was determined based on the inhibitory capacity of Hep at different concentrations from 0 to 0.2 μg) with VAR2HP (0.2 μg) at room temperature for 20 min before added to the wells in the binding assay, The wells adding only VAR2HP were used as positive control. The wells without GAG-Bio were used as the negative control, and the wells with GAG-Bio but without adding recombinant proteins were used as the blank. Each experiment was performed in triplicates.

### Binding kinetic analysis

The binding kinetic analysis between VAR2HP/rVAR2 and Hep/HepG2-GAGs was performed by using a BIAcore T200 system (GE Healthcare). Hep and HepG2-GAGs purified as described above were immobilized on a senor chip as previously reported, respectively (30). Briefly, various concentrations of VAR2HP (16–500 nM) or rVAR2 (32–1000 nM) were injected onto the Hep- or HepG2-GAGs-modified chip surface in running buffer (HBS-EP, pH 7.4, GE Healthcare) with a medium flow rate (30 μl/min) for 90 s as per the manufacturer’s directions. The equilibrium and kinetic constants were calculated by a BIAcore T200 Evaluation Software using 1:1 Langmuir binding model with global fit parameters.

### Extraction of GAGs from cells and tissues

GAGs from cells were extracted as previously described (21) with slight modification. Briefly, cells were delipidated and dehydrated with acetone and ethanol sequentially after collected by cell scraper. The dried powder was digested with heated-activated (60 °C, 30 min) actinase E (2% of dry weight) in 0.1 M sodium borate, 10 mM calcium acetate pH 8.0 at 60 °C for 72 h. Then, the undegraded proteins were removed by precipitation with 5% trichloroacetic acid at ice-bath for 30 min following by centrifugation. The supernatant was extracted with ether to remove trichloroacetic acid and immediately neutralized using 1.0 M sodium carbonate. Then, crude GAGs in the supernatant were precipitated by 80% ethanol containing 5% sodium acetate (w/v) at 4 °C overnight, desalted on a Sephadex G-10 column (GE Healthcare) eluted with 50 mM pyridine acetate, pH 5.0, and freeze-dried. For extraction of GAGs from organs, the organs were first grinded and crushed by adding liquid nitrogen, and then delipidated, dehydrated, and deproteinized as above described.

### Disaccharide composition assay

Disaccharide compositions of various GAGs were analyzed by high HPLC as described previously (21). Briefly, to determine the disaccharide composition of commercial Hep, HS, or Hep/HS in the GAGs extracted from various cells or organs the GAG preparations obtained above were individually digested with by Hepases (I, II, and III) followed by labeling with 2-aminobenzamide. The labeled samples were analyzed by anion exchange HPLC on a YMC-Pack Polyamine II column (YMC-Pack) eluted with a linear gradient from 16 mM to 550 mM NaH_2_PO_4_ over 60 min at a flow rate of 1.0 ml/min at room temperature using a fluorescence detector with excitation and emission wavelengths of 330 and 420 nm, respectively. In the case of CS/DS disaccharide assay, CS/DS sample was digested with chondroitinase ABC (CSase ABC) followed by 2-aminobenzamide labeling and analyzed by anion exchange HPLC on a YMC Pack PA-G column as described above. The percentage of each component was calculated based on its peak area.

### Separation of Hep decasaccharide subfractions in which a fraction P10-7 was preliminarily sequenced

Size-defined unsaturated Hep decasaccharide (UDP10) was further subfractionated using anion exchange HPLC on a Propac PA1 column with a gradient from 1 M to 1.5 M NaCl in 80 min by monitoring at 232 nm. The main peaks (P10-1 to P10-11) were collected and desalted through HPLC on a Superdex Peptide 10/300 GL column at monitoring at 232 nm. The purified components were performed competitive inhibition binding assay by “binding assay” to compare the binding ability with VAR2HP. Furthermore, a fraction P10-7, which has the lowest anion strength among the subfractions with similar binding ability to VAR2HP as Hep polysaccharide, was sequenced by an exo-type Hepase (BIexoHep) as reported previously (33). Briefly, the target fraction (0.5 nM) was partial digestion by BIexoHep (50 mU) for 5 min at 30 °C. The partial digests octasaccharide (P10-7-UDP8), hexasaccharide (P10-7-UDP6), and tetrasaccharide (P10-7-UDP4) were collected on a Superdex Peptide 10/300 GL column eluted with 0.2 M NH_4_HCO_3_ on HPLC detected at 232 nm, respectively, and then were freeze-dried three times to remove NH_4_HCO_3_. The disaccharide compositions of the partial digests and P10-7 were individually determined by Hepases digestion combined with HPLC analysis as described above, and the sequence of P10-7 was deduced based these results.

### Cell binding assay

In this assay, VAR2HP was first biotinylated with EZ-Link Biotin-LC-Hydrazide using the method reported previously (37), and then the VAR2HP-Bio was used as probe to detect the VAR2HP-binding capacities of various cells after the selectivity of VAR2HP-Bio to Hep was determined by binding assay. After optimization of the experimental conditions including VAR2HP concentration (0–4 μg/ml) and blocking agents (BSA, 1%–5%, skim milk, 1%–5%) by 4T1 cells, cells were grown on 96-well plates until they covered the bottom of wells to 80%, fixed with 4% paraformaldehyde, and blocked with 1% BSA for 2 h. Meanwhile, VAR2HP-Bio (0.15 μg) was preincubated with different GAGs with increasing concentrations from 0.2 to 10 μg/ml at room temperature for 20 min, and then the mixtures were added to the cell-immobilized wells for 1.5 h. After washed with PBS three times, the bound protein was detected by HRP-conjugated streptavidin and 3,3′,5,5′-tetramethyl benzidine as a substrate. The wells were analyzed at 450 nm using microplate reader (BioTEK). Each experiment was performed in quintuplicates. The wells without VAR2HP-Bio were used as the blanks, and the wells with VAR2HP-Bio but not preincubated with GAGs were used as positive controls.

To further verify VAR2HP bind to Hep on the cell surface, the cells was treated with or without 5 mU CSase ABC or/and Hepases before added VAR2HP-Bio and then performed the cell binding assay.

### Flow cytometry

To conveniently detect the binding of VAR2HP to living cells by flow cytometry, according to product manual (Sigma) VAR2HP and BSA (as negative control) were labeled with FITC in a molar dye-to-protein (F/P) about 1.0, which was confirmed by analyzing the absorbances at 280 nm and 495 nm by NanoDrop (ThermoFisher). The selectivity of the FITC-labeled VAR2HP (VAR2HP-FITC) was determined by binding assay with a minor modification. Briefly, wells coated with various GAGs were incubated with 50 μl VAR2HP-FITC (0.01 μg/μl) for 30 min in the dark. After washing, the fluorescence intensities of wells were detected by a microplate reader (BioTEK) at excitation 480 nm and emission 520 nm.

To detect and quantify the binding of VAR2HP to living cells, different types of cells were collected after detached with 0.02% EDTA in PBS solution and blocked by 1% BSA for 30 min at room temperature. Then, the cells were incubated with 100 μl VAR2HP-FITC or BSA-FITC (5 μg) in PBS, incubated on ice for 20 min, washed three times by PBS, and then analyzed on a FACS Cytometer (BD Biosciences). To investigate whether VAR2HP selectively binds to Hep/HS chains on cell surface, cells were pretreated with CSase ABC (5 mU) or Hepase (I, II, and III, 2 mU each) at 37 °C for 30 min before adding VAR2HP-FITC.

### Emission wavelength scanning

To determine the fluorescence spectral characteristics of VAR2HP conjugated with fluorescent molecule FITC, emission wavelength scanning was performed for FITC (0.01 μg/ml) and VAR2HP-FITC (2 μg/ml) under excitation at 480 nm by using a fluorescence measurement (HITACHI F-4600, FL Solutions).

### Cell visualization

To visualize the binding of VAR2HP to cell surface, cells were seeded on poly-L-lysine–treated coverslips until cells covered 80% of coverslip surface and then immobilized with 4% paraformaldehyde, washed by PBS, and treated with or without CSase ABC (5 mU) or Hepases (I, II, and III, 2 mU each) at 37 °C for 30 min. The coverslips were blocked with 1% BSA in PBS for 2 h at room temperature and incubated with 200 μl VAR2HP-Bio (0.1 μg/μl) in PBS for 1.5 h. After washing three times with PBS, the bound VAR2HP and nucleus were stained with streptavidin protein (FITC) and DAPI, respectively, and visualized by a scanning laser microscope LSM700 (Car Zeiss Inc) with a Zeiss LSM image Browser.

### The localization and selectivity analysis of VAR2HP *in vivo*

To detect the biocompatibility and localization of VAR2HP *in vivo*, VAR2HP-FITC (20 mg/kg) was injected into 6 weeks Balb/c female mice *via* tail vain. The mice were euthanized at time intervals ranging from 10 min to 8 h. BSA-FITC (20 mg/kg) was injected as negative group. The organs of the mice were removed and photographed by a plant living imagine system (Lumazone pylon 2048B, Roper Scientific).

To further verify the selectivity of VAR2HP to HS/Hep *in vivo*, Hepases (I, II, and III, 70 mU each/mouse) or CSase ABC (200 mU/mouse) were injected into mice *via* tail veins with PBS as negative control. After 1 h, each mouse was injected VAR2HP-FITC at dose of 20 mg/kg. After another 30 min, the organs were taken out and photographed as above described.

To evaluate the targeting of VAR2HP-FITC to tumors *in vivo*. Tumor-bearing mouse were first prepared as reported previously (30). Briefly, 4T1 cells in exponential growth phage were harvested after digested by 0.02% EDTA in PBS for 3 min. The 4T1 cells were injected into mice through tail vain (1 × 10^6^ cells per mouse). After about 20 days, VAR2HP-FITC was injected to the tumor-bearing mice *in vivo* by tail veins at dose of 20 mg/Kg. PBS and BSA-FITC were injected as blank and negative controls, respectively. After 30 min, the mice were photographed using an IVIS Spectrum System and Living Image Software (PerkinElmer) to assess fluorescence intensity distribution of VAR2HP-FITC in tissue. Finally, the mice were euthanized, and their tumorigenic sites were photographed by a gel imaging system (VILBER FUSION FX6 EDGE, Evolution-Capt). Each experiment was done three times in parallel at least.

### Statistic and reproducibility

Statistical analyses were performed using Origin 8.0. Each experiment was done at least three times by triplicates and quintuplicates for cell binding experiments. For comparison of the statistical differences between tween groups, Student’s t-text (two-text) was carried out.

## Data availability

All data supporting the findings of this study are available within the paper (and its Supplementary Information files). All relevant data generated during this study or analyzed in this published article (and its Supplement files) are available from the corresponding author on reasonable request. Source data are provided with this paper.

## Supporting information

This article contains supporting information (30, 37).

## Conflict interest

The authors declare they have no conflicts of interest with the contents of this article.
