# Supplementary material for: A mutated glycosaminoglycan-binding domain functions as a novel probe to selectively target heparin-like epitopes on tumor cells
Source: J Biol Chem. 2022 Oct 17;298(12):102609. doi: 10.1016/j.jbc.2022.102609 (PMC9672413; doi:10.1016/j.jbc.2022.102609)
Supplement: Supporting Information [file mmc1.docx]

Supporting Information

**A mutated glycosaminoglycan-binding domain functions as a novel probe to selectively target heparin-like epitopes on tumor cells**

Yingying Xu^1^, Liran Shi^1,2^, Yong Qin^1^, Xunyi Yuan^3^, Xu Wang^1^, Qingdong Zhang^1,4^, Lin Wei^1^, Min Du^1^, Yi Liu^5^, Min Yuan^1^, Xiangyu Xu^1^, Ruiqing Cheng^1^, Ruyi Zou^1^, Wenshuang Wang^1,*^ and Fuchuan Li^1,*^

**Author’s Affiliation**

^1^National Glycoengineering Research Center and Shandong Provincial Key Laboratory of Carbohydrate Chemistry and Glycobiology, Shandong University; ^2^CSPC Megalith Biopharmaceutical Co., Ltd.; ^3^Department of General Surgery, Qilu Hospital, Shandong University; ^4^School of Life Science and Technology, Weifang Medical University and ^5^ Department of Gastroenterology, the Affiliated Hospital of Qingdao University, Shandong, China.

**Corresponding author**

*(F. Li) Email: fuchuanli@sdu.edu.cn. Tel: +86-532-58631406 Fax: +86-532-58631405.

*(W. Wang) Email: wenshuangwang@sdu.edu.cn. Tel: +86-532-58631406 Fax: +86-532-58631405.

**Supplemental** **Figures and Tables**

**Table S1.** The disaccharide compositions of GAGs used in this study.

|  |  | **CS-A** | **CS-C** | **CS-E** | **DS** |
| --- | --- | --- | --- | --- | --- |
|  | **Unsaturated disaccharide** | **2-AB labeled disaccharide (percentage) mol%** | | | |
| **Unsaturated disaccharide of CS/DS** | ΔHexA(1-3)GalNAc | 1.10 | -- ^a^ | 7.10 | 2.48 |
|  | ΔHexA(1-3)GalNAc4S | 80.87 | 36.78 | 35.80 | 97.52 |
|  | ΔHexA(1-3)GalNAc6S | 18.03 | 45.20 | 12.60 | -- |
|  | ΔHexA2S(1-3)GalNAc6S | -- | 18.02 | -- | -- |
|  | ΔHexA(1-3)GalNAc(4S, 6S) | -- | -- | 44.50 | -- |
|  | **Source** | **(37)** | **Figure S1** | **(30)** | **(37)** |
| **Unsaturated disaccharide of Hep/HS** |  | **Hep** | | **HS** | |
|  | ΔHexA(1-4)GlcNAc | 7.13 | | 31.72 | |
|  | ΔHexA(1-4)GlcNAc6S | 3.47 | | 16.31 | |
|  | ΔHexA(1-4)GlcNS | 5.89 | | 31.47 | |
|  | ΔHexA(1-4)GlcNS6S | 11.70 | | 3.95 | |
|  | ΔHexA2S(1-4)GlcNS | 6.47 | | 9.48 | |
|  | ΔHexA2S(1-4)GlcNS6S | 65.34 | | 7.08 | |
|  | **Source** | **Figure S1** | | **Figure S1** | |

^a^ Not detected. ΔHexA, unsaturated hexuronic acid; GalNAc, *N*-acetyl-galactosamine; GlcNAc, *N*-acetyl-glucosamine; 2S, 2-*O*-sulfate, 4S, 4-*O*-sulfate, NS, *N*-sulfate, 6S, 6-*O*-sulfate.

**Table S2.** The disaccharide compositions of HepG2-GAGs.

|  | **Unsaturated disaccharide** | **Content (pmol)^a^ [%]^b^/(%)^c^** |
| --- | --- | --- |
| **Unsaturated disaccharide of CS/DS** | ΔHexA(1-3)GalNAc | 18.00 [0.65] (15.89) |
|  | ΔHexA(1-3)GalNAc4S | 56.58 [2.06] (49.96) |
|  | ΔHexA(1-3)GalNAc6S | 23.91 [0.87] (21.11) |
|  | ΔHexA2S(1-3)GalNAc6S | 14.76 [0.54] (13.03) |
|  | ΔHexA(1-3)GalNAc(4S, 6S) | --/--^d^ |
| **Total content of CS/DS** |  | 113.35 [**4.12**] (100) |
| **Unsaturated disaccharide of Hep/HS** | ΔHexA(1-4)GlcNAc | 1044.31 [37.96] (39.59) |
|  | ΔHexA(1-4)GlcNAc6S | 254.48 [9.25] (9.65) |
|  | ΔHexA(1-4)GlcNS | 804.97 [29.26] (30.52) |
|  | ΔHexA(1-4)GlcNS6S | 105.64 [3.84] (4.00) |
|  | ΔHexA2S(1-4)GlcNS | 247.87 [9.01] (9.40) |
|  | ΔHexA2S(1-4)GlcNS6S | 180.47 [6.56] (6.84) |
| **Total content of Hep/HS** |  | 2637.74 [**95.88**] (100) |
| **Total content of HepG2-GAGs** |  | 2750.99 [100] |

^a^ The amount in 1 mg of dry cells delipidated and dehydrated with acetone and ethanol respectively.

^b^ The proportion of disaccharide residues in the total GAGs of HepG2-GAGs.

^c^ The proportion of disaccharide residues of CS/DS or Hep/HS, respectively.

^d^ Not detected.

**Note:** the proportion of disaccharide units was calculated based on their peak area of HPLC results in Figure S3A.

**Table S3.** The primer pairs of recombinant proteins.

| **Mutants** | **Primer sequence** | **Template** | **DNA polymerase** |
| --- | --- | --- | --- |
| VAR2HP | 5’-GAGCTCCATATGAATTACATCAAAG-3’ | pET22b-ID2a-ID2b | Taq^TM^ polymerase |
|  | 5’-ACTAGTGTGGTGGTGGTGGTGGTGCTCGAGATCCAGTTTGCTGC-3’ |  |  |
| VAR2HP-T | 5’-CATATGGAGAAGTTTCTGGCCGGCTG-3’ | pET22b-var2hp | Phanta Max Super-Fidelity DNA Polymerase |
|  | 5’-CTCGAGGTTCTCCACCCATTCTTGGA-3’ |  |  |
| VAR2HP-noAsn | 5’-CTGTTTGGTAAGTATATCAAGAAAAATAACACGGCGG-3’ | pET22b-var2hp | Phanta Max Super-Fidelity DNA Polymerase |
|  | 5’-TATACTTACCAAACAGTTTGCCAAAATTATTCTGGAG-3’ |  |  |
| TP-Asn | 5’-CTGTTTGGGAATAAGTATATCAAGAAAAATAACACGGCGG-3’ | pET22b-ID2a-ID2b | Phanta Max Super-Fidelity DNA Polymerase |
|  | 5’-TATACTTATTCCCAAACAGTTTGCCAAAATTATTCTGGAG-3’ |  |  |

**Note:** The underline in primers were Sac I and Spe I recognition sites, respectively**,** the double underline in primers were Nde I and Xho I recognition sites, respectively. The usage of DNA polymerase was followed its manufacturer’s instructions.

**Table S4.** Disaccharide compositions of size-defined heparin oligosaccharides.

|  | **Hep UDP2** | | | **Hep UDP4** | | **Hep UDP6** | **Hep UDP8** | | **Hep UDP10** | | **Hep UDP12** | | **Hep UDP14** | | **Hep UDP16** | | | **Hep** |
| --- | --- | --- | --- | --- | --- | --- | --- | --- | --- | --- | --- | --- | --- | --- | --- | --- | --- | --- |
| **Disaccharide units** | | **Disaccharide units (%)** | | | | | | | | | | | | | | | |  |
| ΔHexA(1-4)GlcNAc | | 0.24 | | 1.72 | | 2.99 | 1.73 | | 1.59 | | 1.96 | | 1.04 | | 1.27 | | | 7.13 |
| ΔHexA(1-4)GlcNAc6S | | 1.27 | | 0.99 | | 10.52 | 7.36 | | 4.9 | | 4.13 | | 2.69 | | 2.57 | | | 3.47 |
| ΔHexA(1-4)GlcNS | | 1.68 | | 10.17 | | 16.83 | 14.3 | | 10.95 | | 7.68 | | 8.82 | | 6.18 | | | 5.89 |
| ΔHexA(1-4)GlcNS6S | | 5.72 | | 33.59 | | 11.80 | 13.85 | | 13.87 | | 11.99 | | 11.47 | | 9.06 | | | 11.70 |
| ΔHexA2S(1-4)GlcNS | | 3.97 | | 5.19 | | 7.43 | 8.71 | | 8.31 | | 9.51 | | 7.75 | | 9.92 | | | 6.47 |
| ΔHexA2S(1-4)GlcNS6S | | 87.12 | | 48.34 | | 50.43 | 54.05 | | 60.38 | | 64.73 | | 68.23 | | 71.00 | | | 65.34 |
| **Total (%)** | | 100 | 100 | | 100 | | | 100 | | 100 | | 100 | | 100 | | 100 | 100 | |

**Note:** the proportion of disaccharide units was calculated based on their peak area of HPLC results in Figure S1 and S6.

**Table S5.** Disaccharide compositions of subfractions of Hep UDP 10.

|  | **P10-1** | | **P10-2** | **P10-3** | **P10-4** | **P10-5** | **P10-6** | **P10-7** | **P10-8** | **P10-9** | **P10-10** | **P10-11** |
| --- | --- | --- | --- | --- | --- | --- | --- | --- | --- | --- | --- | --- |
| **Disaccharide units** | | **Disaccharide units (%) [molecular ratio** ^a^**]** | | | | | | | | | | |
| ΔHexA(1-4)GlcNAc | | --/-- | --/-- | --/-- | --/-- | --/-- | --/-- | --/-- | --/-- | --/-- | --/-- | --/-- |
| ΔHexA(1-4)GlcNAc6S | | 11.23[0.6] | 4.54[0.2] | --/-- | --/-- | --/-- | --/-- | --/-- | --/-- | --/-- | --/-- | --/-- |
| ΔHexA(1-4)GlcNS | | 17.95[0.9] | 22.13[1.1] | 9.34[0.5] | 14.42[0.7] | 11.07[0.6] | 10.79[0.5] | 12.7[0.6] | --/-- | --/-- | --/-- | --/-- |
| ΔHexA(1-4)GlcNS6S | | 13.09[0.7] | 17.49[0.9] | 20.66[1.0] | 16.07[0.8] | 20.52[1.0] | 9.73[0.5] | 14.07[0.7] | 25.66[1.3] | 14.91[0.7] | 19.95[1.0] | 12.04[0.6] |
| ΔHexA2S(1-4)GlcNS | | 11.98[0.6] | 9.92[0.5] | 14.50[0.7] | 8.57[0.4] | 6.02[0.3] | 13.45[0.7] | 3.61[0.2] | 8.00[0.4] | 5.26[0.3] | --/-- | --/-- |
| ΔHexA2S(1-4)GlcNS6S | | 45.75[2.3] | 45.92[2.3] | 55.50[2.8] | 60.94[3.0] | 62.39[3.1] | 66.03[3.3] | 69.62[3.5] | 66.34[3.3] | 79.83[4.0] | 80.05[4.0] | 87.96[4.4] |
| **Total (%)** | 100[5.0] | | 100[5.0] | 100[5.0] | 100[5.0] | 100[5.0] | 100[5.0] | 100[5.0] | 100[5.0] | 100[5.0] | 100[5.0] | 100[5.0] |
| **Sulfation degree**^b^ | 11.0 | | 11.0 | 12.3 | 12.1 | 12.5 | 13.8 | 12.9 | 13.3 | 14.0 | 14.0 | 14.4 |

^a^ The proportion of the disaccharide residue in the decasaccharide sequence, which was calculated by dividing the proportion of the disaccharide residue by 20%.

^b^ The average of sulfation degree of a decasaccharide.

**Table S6.** Disaccharide compositions of HS chains on the surface of various cells.

|  | **HuH-7** | **A549** | **HeLa** | **Hep3B** | **4T1** | **Li-7** | **HT29** | **293T** |  |
| --- | --- | --- | --- | --- | --- | --- | --- | --- | --- |
| **Disaccharide units** | **Content (pmol) [%]** | | | | | | | | |
| ΔHexA(1-4)GlcNAc | 675.81[40.01] | 453.21[40.69] | 549.52[49.07] | 395.20[47.59] | 303.39[51.70] | 223.86[52.07] | 145.77[57.41] | 64.07[38.98] |  |
| ΔHexA(1-4)GlcNAc6S | 261.30[15.47] | 63.93[5.74] | 114.56[10.23] | 143.33[17.26] | 13.97[2.38] | 27.56[6.41] | 20.26[7.98] | 26.56[16.16] |  |
| ΔHexA(1-4)GlcNS | 315.19[18.66] | 354.75[31.85] | 233.94[20.89] | 109.03[13.13] | 186.49[31.78] | 116.64[27.13] | 54.81[21.59] | 36.26[22.06] |  |
| ΔHexA(1-4)GlcNS6S | 181.24[10.73] | 60.59[5.44] | 71.78[6.41] | 111.44[13.42] | 15.26[2.60] | 3.44[0.80] | 10.99[4.33] | 14.59[8.88] |  |
| ΔHexA2S(1-4)GlcNS | 47.13[2.79] | 94.78[8.51] | 61.03[5.45] | --/--^b^ | 39.49[6.73] | 27.77[6.46] | 13.38[5.27] | 13.46[8.19] |  |
| ΔHexA2S(1-4)GlcNS6S | 208.43[12.34] | 86.54[7.77] | 89.03[7.95] | 71.42[8.60] | 28.23[4.81] | 30.69[7.14] | 8.68[3.42] | 9.42[5.73] |  |
| **Total^a^ (pmol) [%]** | 1689.10[100] | 1113.80[100] | 1119.86[100] | 830.42[100] | 586.83[100] | 429.96[100] | 253.89[100] | 164.36[100] |  |

^a^ The amount in 1 mg of dry cells delipidated and dehydrated with acetone and ethanol respectively.

^b^ Not detected.

**Note:** the proportion of disaccharide units was calculated based on their peak area of HPLC results in Figure S13.

**Table S7.** Disaccharide compositions of HS/Hep in organs.

|  | **Intestines** | **Liver** | **Kidney** | **Stomach** | **Spleen** | **Lung** | **Heart** |
| --- | --- | --- | --- | --- | --- | --- | --- |
| **Disaccharide unit** | **Content (pmol) [%]** | | | | | | |
| ΔHexA(1-4)GlcNAc | 478.39[59.86] | 192.12[54.51] | 241.81[46.57] | 177.39[46.71] | 150.87[55.40] | 197.73[61.03] | 206.77[62.69] |
| ΔHexA(1-4)GlcNAc6S | 44.35[5.55] | 28.87[8.19] | 52.96[10.20] | 21.31[5.61] | 35.65[13.09] | 14.84[4.58] | 21.77[6.60] |
| ΔHexA(1-4)GlcNS | 149.85[18.75] | 58.12[16.49] | 116.73[22.48] | 95.28[25.09] | 38.94[14.30] | 60.33[18.62] | 59.17[17.94] |
| ΔHexA(1-4)GlcNS6S | 24.69[3.09] | 12.94[3.67] | 36.24[6.98] | 11.01[2.90] | 17.54[6.44] | 5.73[1.77] | 4.78[1.45] |
| ΔHexA2S(1-4)GlcNS | 58.98[7.38] | 23.93[6.79] | 32.87[6.33] | 49.67[13.08] | 13.43[4.93] | 31.98[9.87] | 26.88[8.15] |
| ΔHexA2S(1-4)GlcNS6S | 42.92[5.37] | 36.48[10.35] | 38.63[7.44] | 25.11[6.61] | 15.90[5.84] | 13.38[4.13] | 10.46[3.17] |
| **Content (pmol)**^a^ **[%]** | 799.18[100] | 352.46[100] | 519.24[100] | 379.77[100] | 272.33[100] | 323.99[100] | 329.83[100] |

^a^ The amount in 1 mg of dry tissues delipidated and dehydrated with acetone and ethanol respectively.

**Note:** the proportion of disaccharide units was calculated based on their peak area of HPLC results in Figure S14.


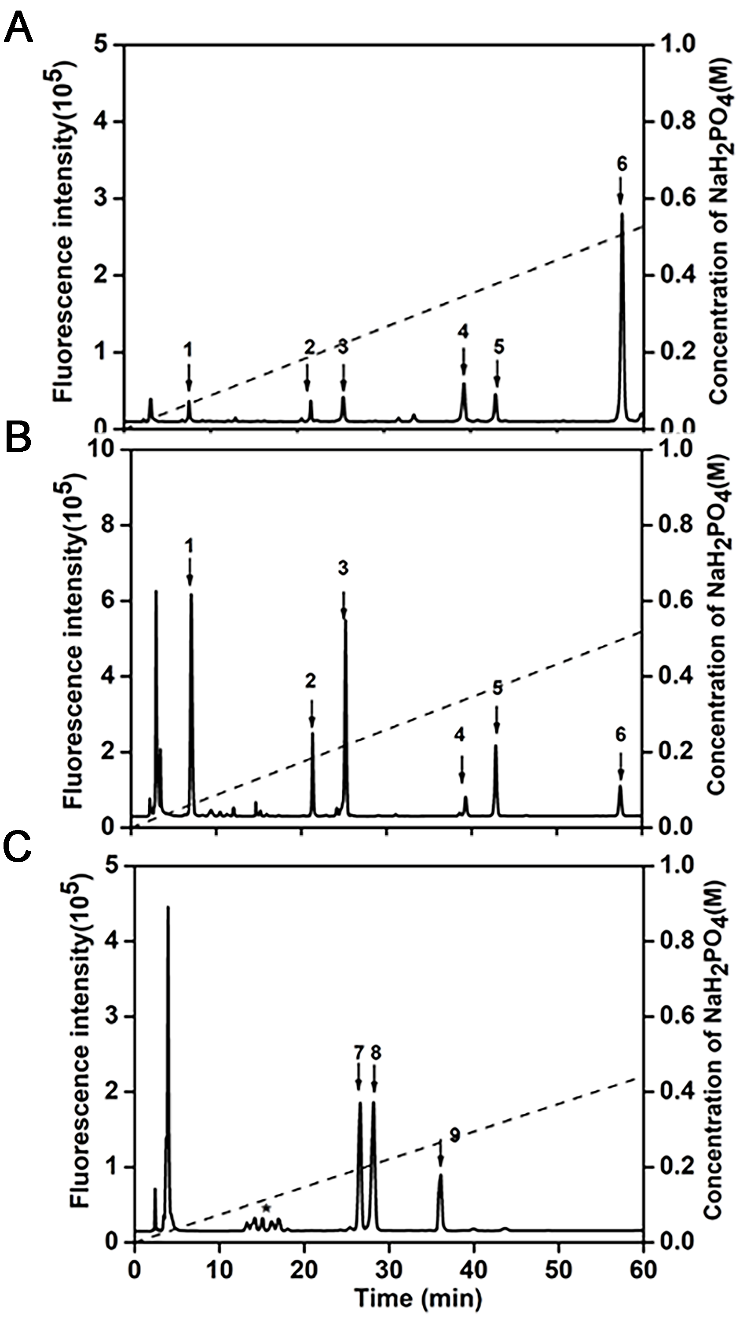


**Figure S1.** The disaccharides compositions of Hep (A), HS (B), CS-C (C). Hep and HS were completely digested with Hepases and CS-C was completely digested with CSase ABC. Then the digests were labeled with 2-AB followed by anion-exchange HPLC analysis with monitoring at excitation 330 nm and emission 420 nm. 1, ΔHexA(1-4)GlcNAc, 2, ΔHexA(1-4)GlcNAc6S, 3, ΔHexA(1-4)GlcNS, 4, ΔHexA(1-4)GlcNS6S, 5, ΔHexA2S(1-4)GlcNS, 6, ΔHexA2S(1-4)GlcNS6S, 7, ΔHexA(1-3)GalNAc4S, 8, ΔHexA(1-3)GalNAc6S, 9, ΔHexA2S(1-3)GalNAc6S. *, impurity peak, the position of the non-sulfated disaccharide unit ΔHexA(1-3)GalNAc of CS was included, but not distinguished due to the low content.


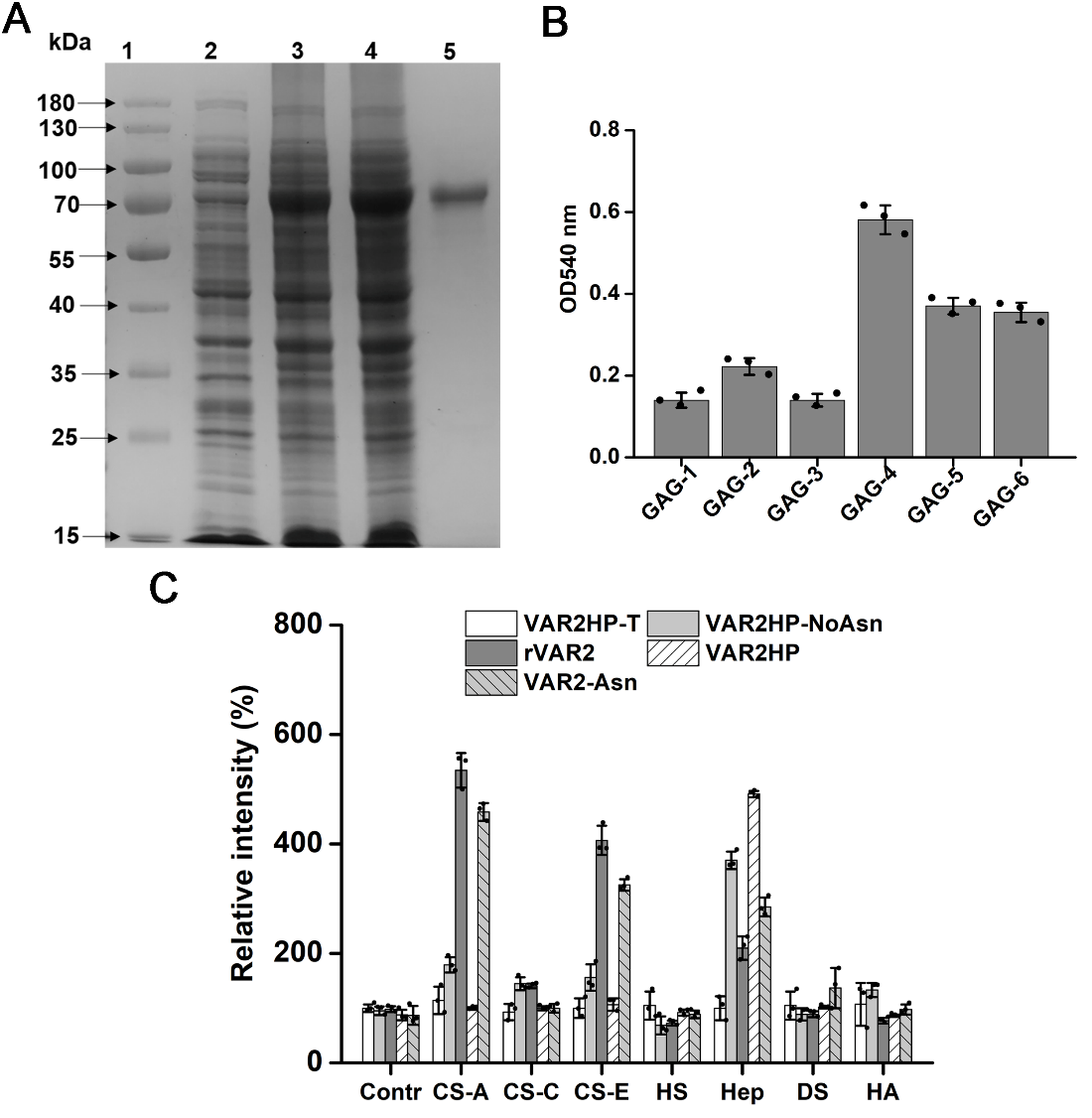


**Figure S2.** (A) Heterologous expression and purification of rVAR2. Recombinant rVAR2 expressed in *E. coli* BL21 (DE3) was purified by nickel affinity chromatography followed by gel filtration and analyzed by SDS-PAGE under reducing conditions. Lane 1, Marker (PageRuler^TM^, ThermoFisher); Lane 2, uninduced cell lysate; Lane 3, induced cell lysate; Lane 4, supernatant fluid of induced cell lysate; Lane 5, purified proteins. (B) Screening of clones for binding to HepG2-GAGs. Six clones with high binding capacities to HepG2-GAGs were screened by a binding assay as described under “*Experimental Procedures*”. (C) Comparison of the binding capacities of recombinant proteins. The data were shown as the relative intensity to that of the control group (Contr) of VAR2HP-T without immobilizing biotinylated GAGs.


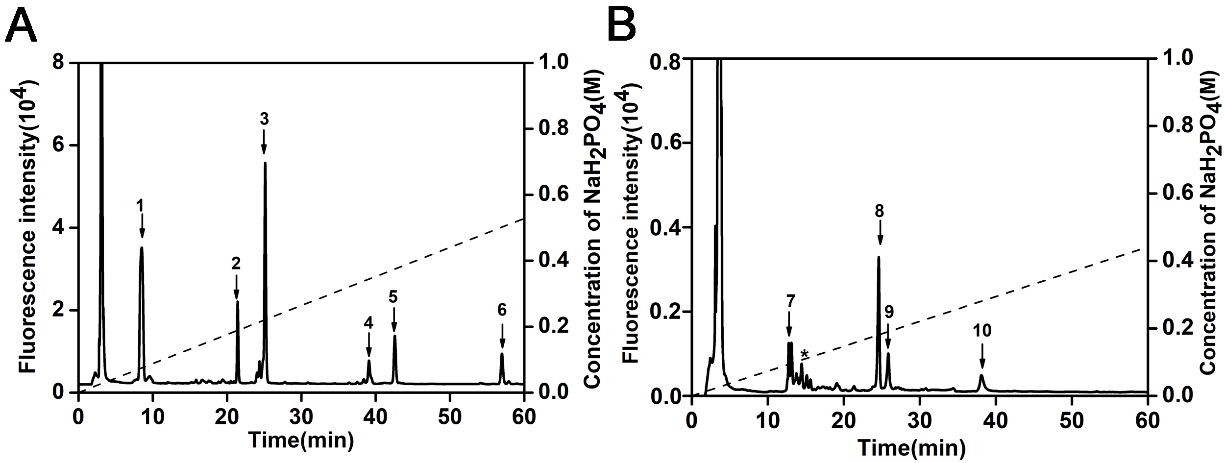


**Figure S3.** Disaccharide analysis of HS (A) and CS/DS (B) in HepG2-GAGs. HepG2-GAGs was completely digested with Hepases (I, II, and III) or CSase ABC, and then labeled with 2-AB followed by anion-exchange HPLC analysis with monitoring at excitation 330 nm and emission 420 nm. 1, ΔHexA(1-4)GlcNAc, 2, ΔHexA(1-4)GlcNAc6S, 3, ΔHexA(1-4)GlcNS, 4, ΔHexA(1-4)GlcNS6S, 5, ΔHexA2S(1-4)GlcNS, 6, ΔHexA2S(1-4)GlcNS6S, 7, ΔHexA(1-3)GalNAc, 8, ΔHexA(1-3)GalNAc4S, 9, ΔHexA(1-3)GalNAc6S, 10, ΔHexA2S(1-3)GalNAc6S. *, impurity peak.


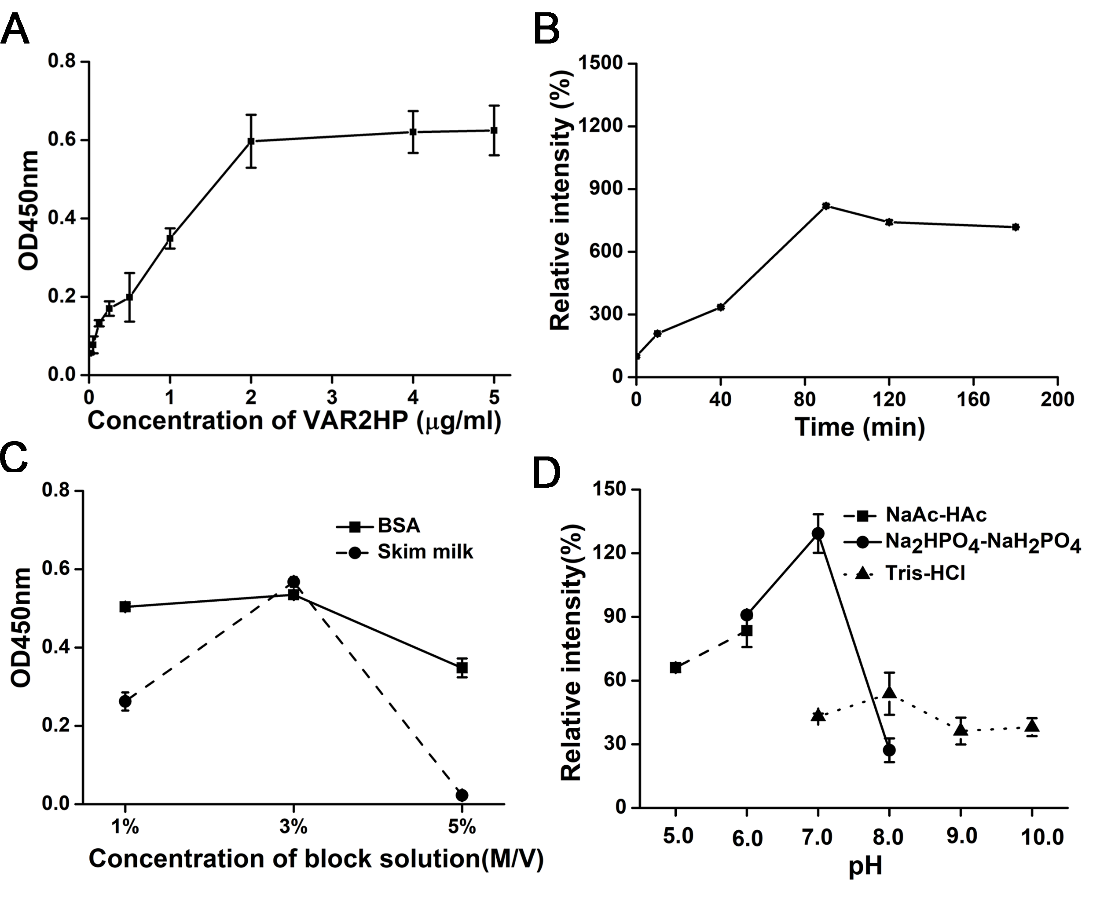


**Figure S4.** Condition optimization for binding assay. The conditions of VAR2HP binding to Hep were optimized under various concentrations of VAR2HP (0-5 µg/ml) (A), incubation time (0-180 min) (B), blocking solutions (1%-5% BSA and skim milk in PBS, respectively) (C), and buffers (10 mM NaAc-HAc, pH 5.0-6.0; 10 mM Na_2_HPO_4_-NaH_2_PO_4_, pH 6.0-8.0; and 10 mM Tris-HCl, pH 7.0-10.0). The results are shown as relative intensity compared to that of wells without VAR2HP in (B), and compared to that of PBS in (D). Error bars represent means of triplicates ±S.D. Based on the results, the optimal conditions of VAR2HP binding to Hep were determined as following: VAR2HP, 4 μg/ml; blocking solution, 3% skim milk; and incubation time, 90 min.


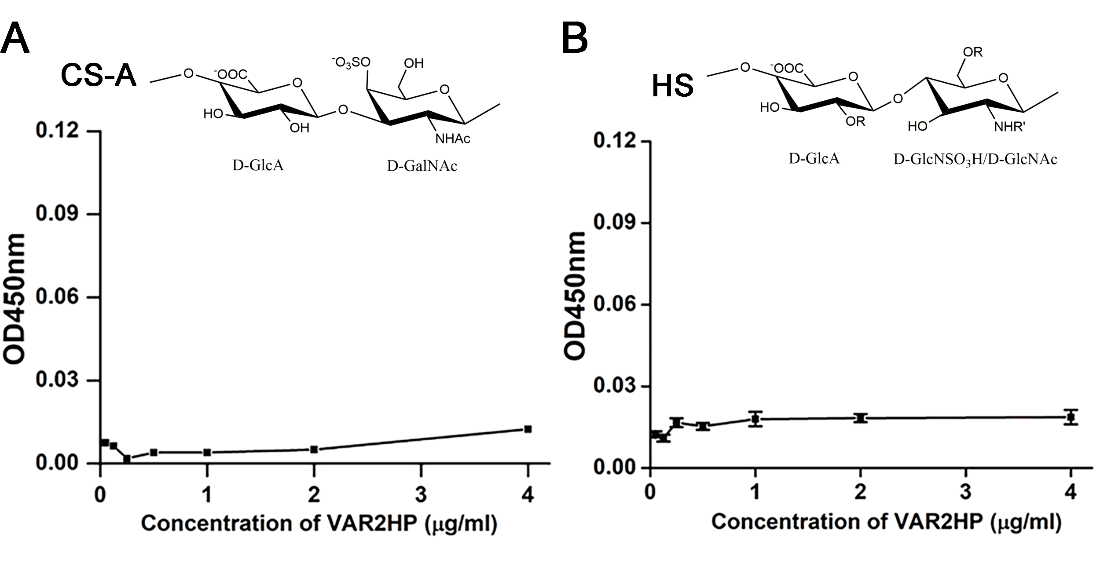


**Figure S5.** Binding capacity of VAR2HP to CS-A (A) and HS (B) with the indicated increasing concentration. Error bars represent means of triplicates ± S.D. *R*: H/SO_3_^-^, *R’*: SO_3_^-^/Ac in (B).


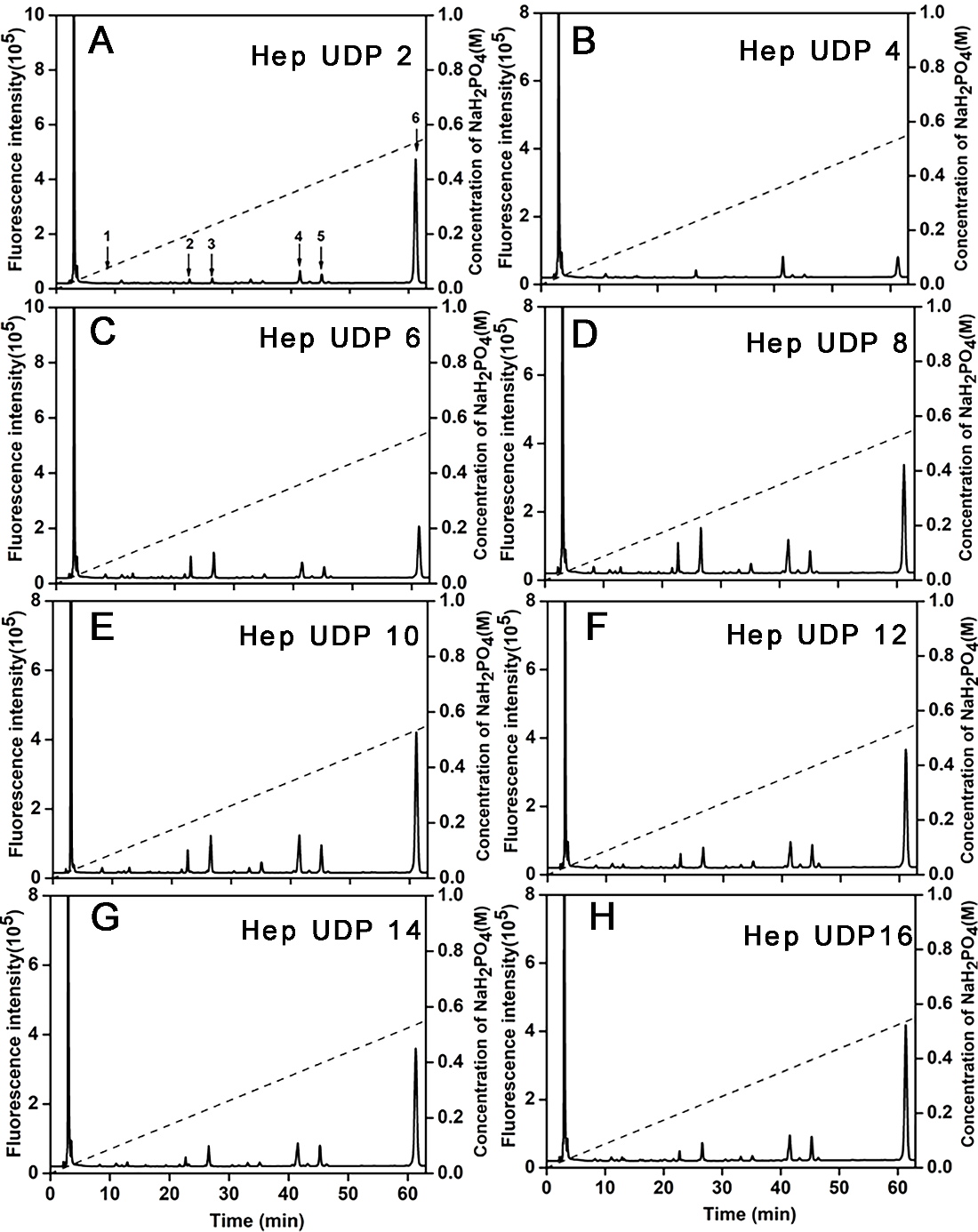


**Figure S6.** Disaccharide composition analysis of size-defined Hep oligosaccharides UDP2 (A), UDP4 (B), UDP6 (C), UDP8 (D), UDP10 (E), UDP12 (F), UDP14 (G), and UDP16 (H). Each oligosaccharide fraction was exhaustively degraded with Hepase (I, II, and III) and labeled with 2-AB for anion-exchange HPLC analysis as described above. 1, ΔHexA(1-4)GlcNAc, 2, ΔHexA(1-4)GlcNAc6S, 3, ΔHexA(1-4)GlcNS, 4, ΔHexA(1-4)GlcNS6S, 5, ΔHexA2S(1-4)GlcNS, 6, ΔHexA2S(1-4)GlcNS6S.


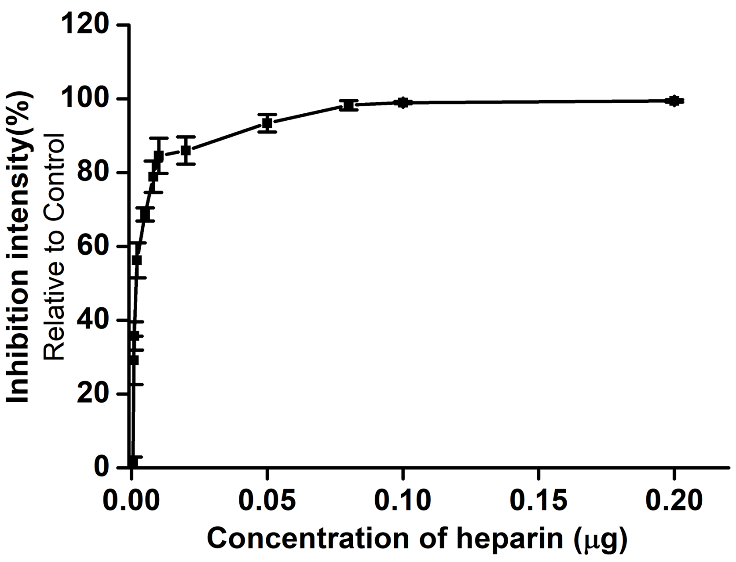


**Figure S7.** Competitive inhibition assay. The binding of VAR2HP to immobilized Hep was competitively inhibited by preincubation with free Hep with increasing concentrations (0-0.20 µg/50 µl). The data are shown as the ratio of the difference absorbance value between the test groups and control group which preincubated with no Hep, to that of the control group. Based on the results, 0.1 µg was chosen as the amounts of inhibitors including Hep polysaccharide and oligosaccharides in the next competitive inhibition assay.


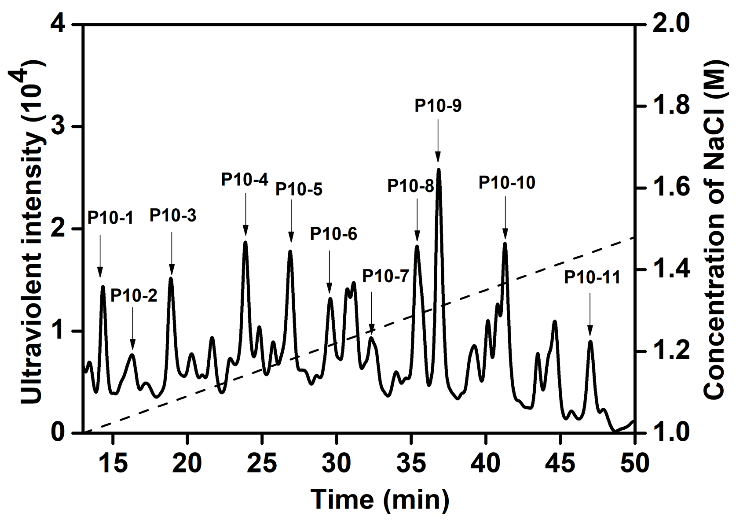


**Figure S8.** Subfractionation of Hep UDP10. Hep decasaccharide UDP10 was separated by anion-exchange HPLC on a Propac PA1 column eluted with a gradient from 1 M to 1.5 M NaCl in 80 min by monitoring at 232 nm.


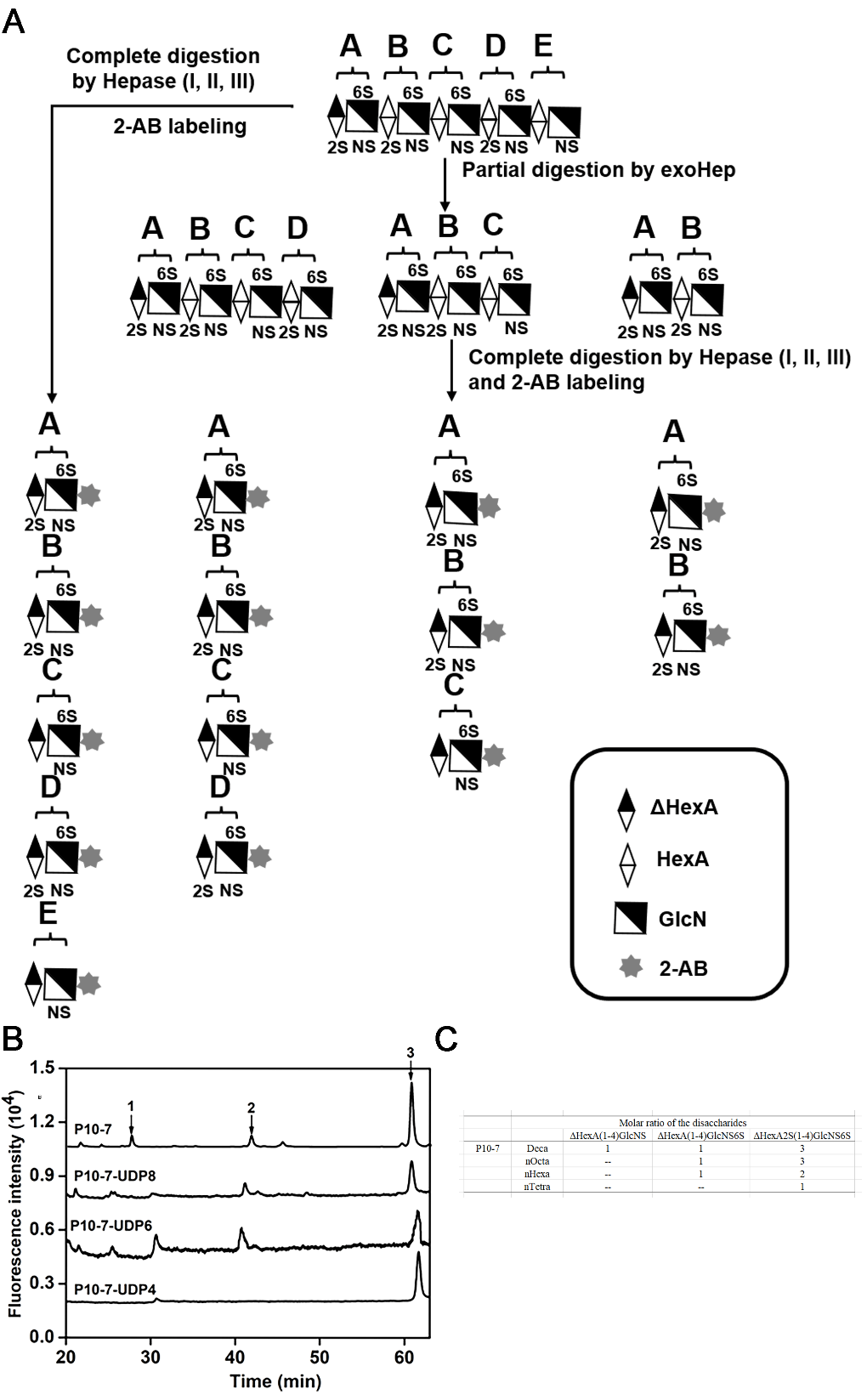


**Figure S9.** Sequencing of P10-7. (A) Strategy for sequencing. (B) Preliminary sequence of P10-7. Decasaccharide P10-7 was partially digested with BIexoHep from reducing end, and resulted octa-, hexa- and tetrasaccharides were separated and collected and completely digested by Hepases for disaccharide composition analysis by anion-exchange HPLC as described above. 1, ΔHexA(1-4)GlcNS, 2, ΔHexA(1-4)GlcNS6S, 3, ΔHexA2S(1-4)GlcNS6S. (C) Molar ratio of the disaccharides in (B).


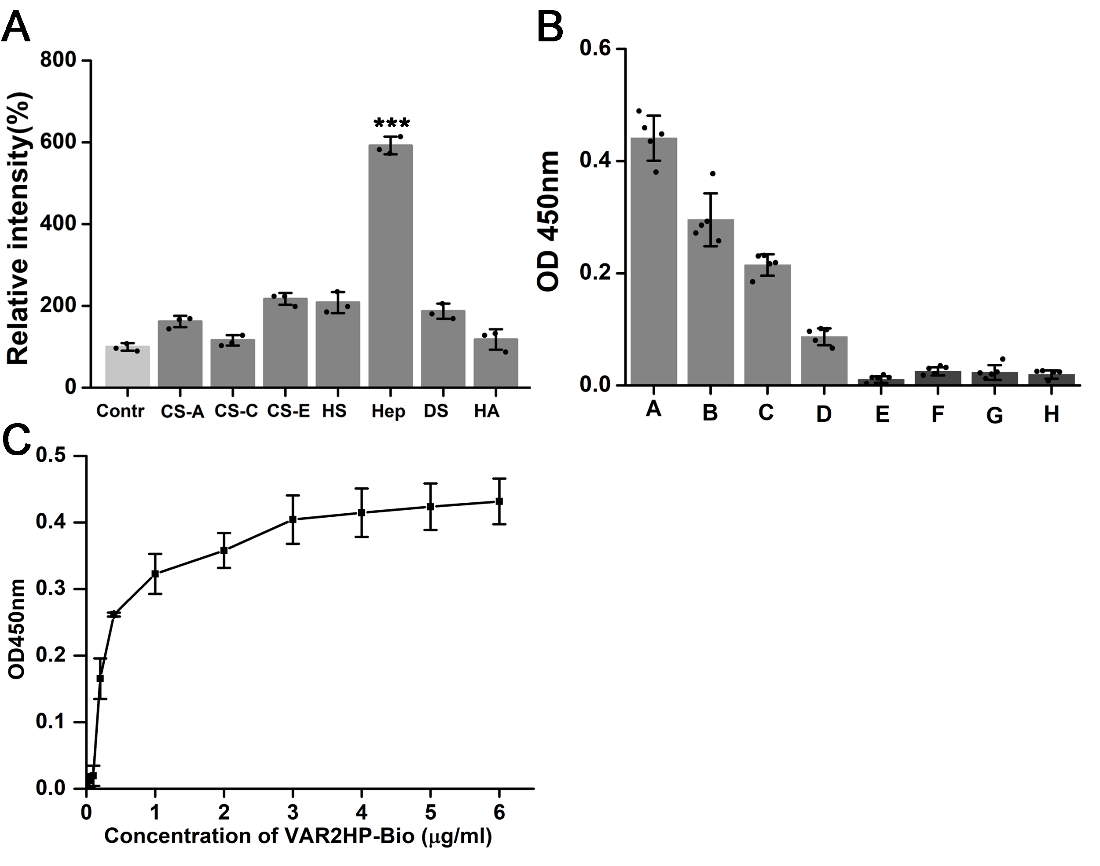


**Figure S10.** Hep-binding specificity and cell-binding conditions of VAR2HP-Bio. (A) Binding capacities of VAR2HP-Bio to various GAGs were determined by binding assay. Data are shown as the percentage of relative intensity related to control without VAR2HP-Bio. p-values in all cases are compared with control (Contr), ***p* <0.01, ****p* *<*0.001. (B) Effects of different blocking solution on the binding of VAR2HP-Bio to 4T1 cells. A-D,1%, 2%, 3%, 5% BSA in PBS; E-H, 1%, 2%, 3%, 5% skim milk in PBS. (C) Effect of concentration of VAR2HP-Bio on binding to 4T1 cells. Error bars represent average of quintuplicates ± S.D. Base on the results, 3 μg/ml VAR2HP-Bio and 1% BSA in PBS were chosen for the next cell binding assay of VAR2HP.


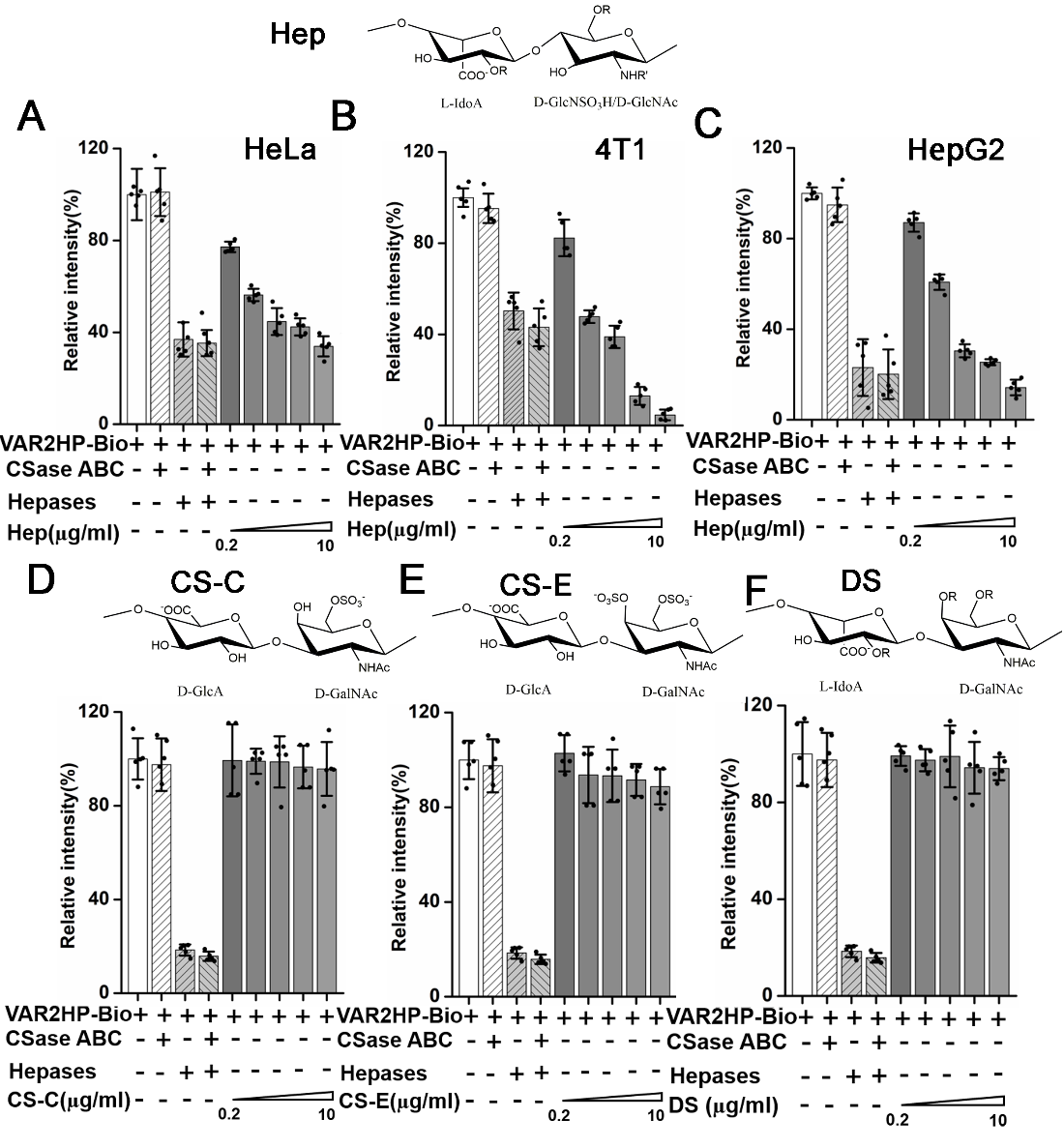


**Figure S11.** Binding assay of VAR2HP-Bio to HeLa cells (A), 4T1 cells (B), HepG2 cells (C) treated with or without Hepases or/and CSase ABC, or HuH-7 cells in the presence of CS-C (D), CS-E (E) and DS (F) at different concentrations (0.2, 2.5 5, 7.5 and 10 µg/ml, respectively). Error bars represent average of quintuplicates ± S.D. *R*: H/SO_3_^-^, *R’*: SO_3_^-^/Ac in figures.


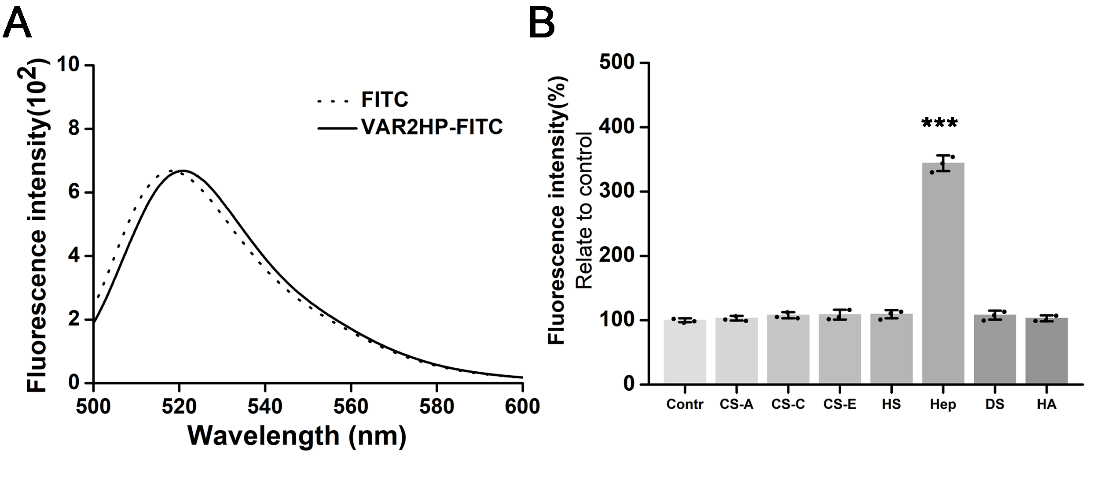


**Figure S12.** Emission wavelength scanning and selectivity of VAR2HP-FITC. (A) The emission wavelength of VAR2HP-FITC and FITC was scanned by a fluorescence measurement (HITACHI F-4600, Japan) under excitation at 480nm. *Black line*, VAR2HP-FITC. *Dot line*, FITC. (B) Binding capacity of VAR2HP-FITC (0.5 µg) to various GAGs were analyzed by binding assay as described above. Error bars represent average of triplicates ± S.D. p- values in all cases are compared with control group (contr) without immobilized GAGs-Bio, ****p* *<*0.001.


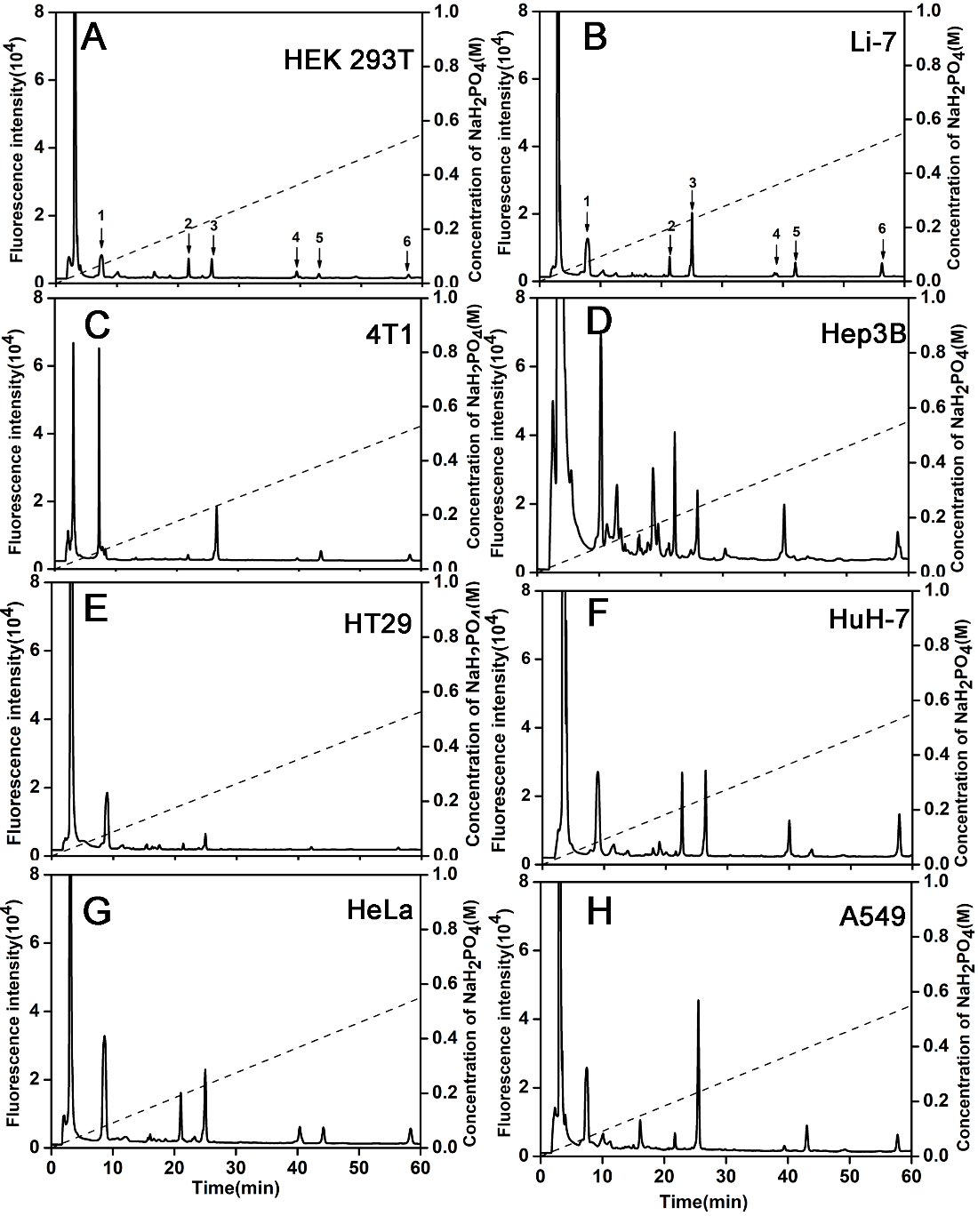


**Figure S13.** The disaccharide composition analysis of HS extracted from HEK 293T (A), Li-7 (B), 4T1 (C), Hep3B (D), HT29 (E), HuH-7 (F), HeLa (G), and A549 (H). GAGs extracted from cells were exhaustively digested with Hepase (I, II, and III), and labeled with 2-AB for anion-exchange HPLC analysis as described above. 1, ΔHexA(1-4)GlcNAc, 2, ΔHexA(1-4)GlcNAc6S, 3, ΔHexA(1-4)GlcNS, 4, ΔHexA(1-4)GlcNS6S, 5, ΔHexA2S(1-4)GlcNS, 6, ΔHexA2S(1-4)GlcNS6S.


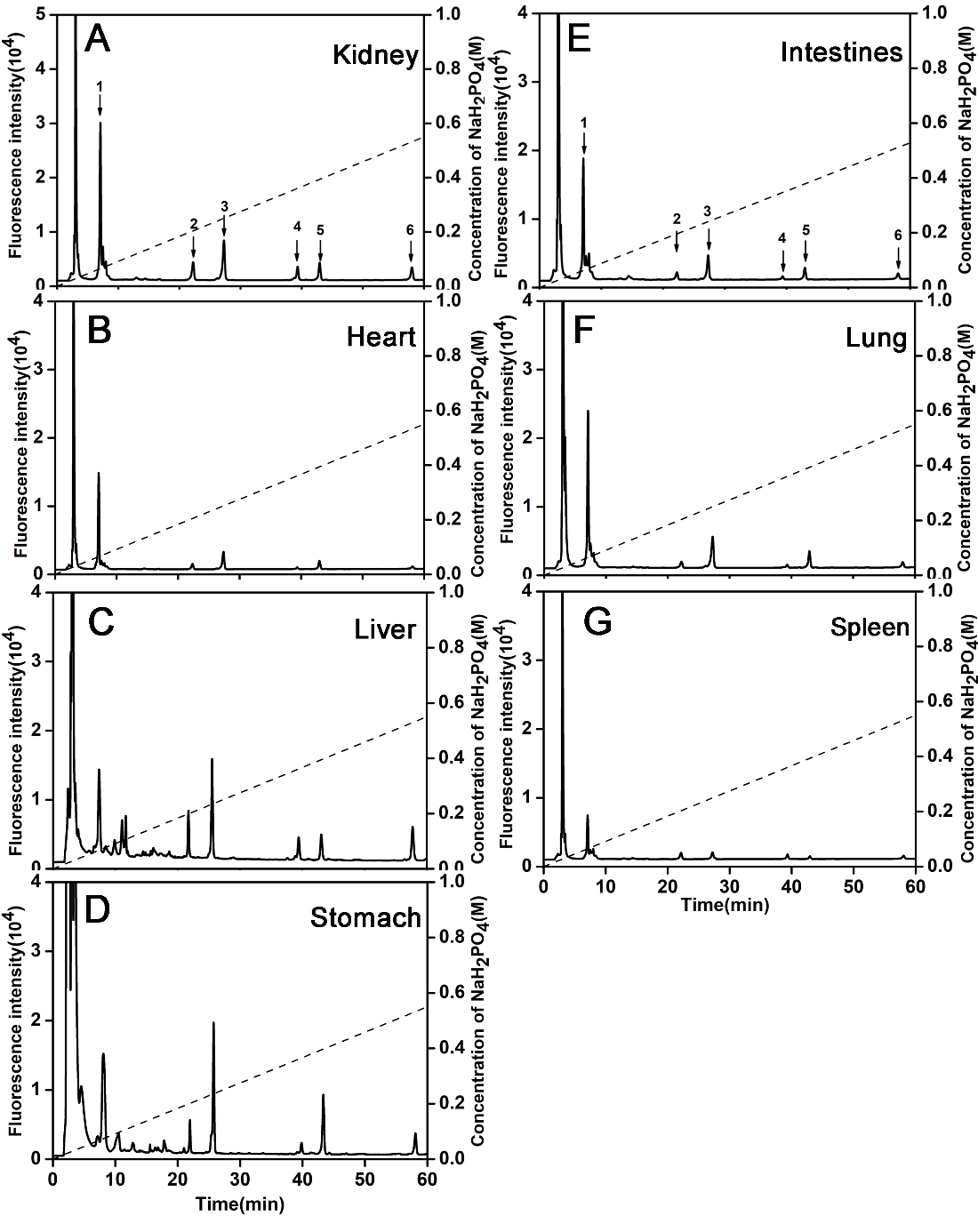


**Figure S14**. The disaccharide composition analysis of HS/Hep from kidney (A), heart (B), liver (C), stomach (D), intestines (E), lung (F), and spleen (G). GAGs extracted from organs were exhaustively digested with Hepase (I, II, and III) and labeled with 2-AB for HPLC analysis as described above. 1, ΔHexA(1-4)GlcNAc, 2, ΔHexA(1-4)GlcNAc6S, 3, ΔHexA(1-4)GlcNS, 4, ΔHexA(1-4)GlcNS6S, 5, ΔHexA2S(1-4)GlcNS, 6, ΔHexA2S(1-4)GlcNS6S.

**References**

30. Peng, C., Wang, Q., Jiao, R., Xu, Y., Han, N., Wang, W., Zhu, C., and Li, F. (2021) A novel chondroitin sulfate E from Dosidicus gigas cartilage and its antitumor metastatic activity. *Carbohydr. Polym.* **262**, 117971

37. Wang, W., Han, N., Xu, Y., Zhao, Y., Shi, L., Filmus, J., and Li, F. (2020) Assembling custom side chains on proteoglycans to interrogate their function in living cells. *Nat. Commun.* **11**, 5915
